# Devices Used to Measure Force-Time Characteristics of Spinal Manipulations and Mobilizations: A Mixed-Methods Scoping Review on Metrologic Properties and Factors Influencing Use

**DOI:** 10.3389/fpain.2021.755877

**Published:** 2021-10-29

**Authors:** Marie-Andrée Mercier, Philippe Rousseau, Martha Funabashi, Martin Descarreaux, Isabelle Pagé

**Affiliations:** ^1^Chiropractic Department, Université du Québec à Trois-Rivières, Trois-Rivières, QC, Canada; ^2^Canadian Memorial Chiropractic College, Toronto, ON, Canada; ^3^Human Kinetics Department, Université du Québec à Trois-Rivières, Trois-Rivières, QC, Canada; ^4^Center for Interdisciplinary Research in Rehabilitation and Social Integration (CIRRIS), Québec, QC, Canada

**Keywords:** spinal manipulation, spinal mobilization, metrologic properties, scoping review, limiting factors, facilitating factors, mixed-methods, force-time characteristics

## Abstract

**Background:** Spinal manipulations (SMT) and mobilizations (MOB) are interventions commonly performed by many health care providers to manage musculoskeletal conditions. The clinical effects of these interventions are believed to be, at least in part, associated with their force-time characteristics. Numerous devices have been developed to measure the force-time characteristics of these modalities. The use of a device may be facilitated or limited by different factors such as its metrologic properties.

**Objectives:** This mixed-method scoping review aimed to characterize the metrologic properties of devices used to measure SMT/MOB force-time characteristics and to determine which factors may facilitate or limit the use of such devices within the context of research, education and clinical practice.

**Methods:** This study followed the Joanna Briggs Institute's framework. The literature search strategy included four concepts: (1) devices, (2) measurement of SMT or MOB force-time characteristics on humans, (3) factors facilitating or limiting the use of devices, and (4) metrologic properties. Two reviewers independently reviewed titles, abstracts and full articles to determine inclusion. To be included, studies had to report on a device metrologic property (e.g., reliability, accuracy) and/or discuss factors that may facilitate or limit the use of the device within the context of research, education or clinical practice. Metrologic properties were extracted per device. Limiting and facilitating factors were extracted and themes were identified.

**Results:** From the 8,998 studies initially retrieved, 46 studies were finally included. Ten devices measuring SMT/MOB force-time characteristics at the clinician-patient interface and six measuring them at patient-table interfaces were identified. Between zero and eight metrologic properties were reported per device: measurement error (defined as validity, accuracy, fidelity, or calibration), reliability/repeatability, coupling/crosstalk effect, linearity/correlation, sensitivity, variability, drift, and calibration. From the results, five themes related to the facilitating and limiting factors were developed: user-friendliness and versatility, metrologic/intrinsic properties, cost and durability, technique application, and feedback.

**Conclusion:** Various devices are available to measure SMT/MOB force-time characteristics. Metrologic properties were reported for most devices, but terminology standardization is lacking. The usefulness of a device in a particular context should be determined considering the metrologic properties as well as other potential facilitating and limiting factors.

## Introduction

Spinal manipulation therapy (SMT) and spinal mobilization (MOB) are characterized by the application of a force whose characteristics vary over the application time (1). As such, SMT and MOB can be described as a function of their biomechanical parameters, including preload force, total peak force, thrust duration and rates of force applications (1). Specific force-time characteristics have been shown to impact the neuromechanical responses triggered during SMT and MOB, such as paraspinal muscle reflexes amplitude, intervertebral displacement and H-reflex characteristics (2, 3). Moreover, the loads sustained by spinal functional unit components have also been shown to vary according to SMT force-time characteristics (4, 5). It has, therefore, been hypothesized that SMT/MOB clinical effects are, at least partly, associated with their force-time characteristics.

To measure the force-time characteristics of SMT and MOB delivered by manual therapists, several research teams have either developed devices or adapted commercially available ones (6). These devices either record forces directly at the clinician's hand (i.e., at the clinician-patient level) or indirectly at the patient-table interface. For instance, pressure pad and small size load sensors have been positioned between the clinician's hand and the patient's back during the application of a thoracic SMT [e.g., Herzog et al. (7) and Kirstukas and Backman (8)]. On the other hand, load cells and force plates have also been mounted between the frame and the cushion of commercial standard treatment tables [e.g., Chiradejnant et al. (9) and Snodgrass et al. (10)]. These devices have been used not only in research to investigate SMT and MOB's biomechanics and training, but also as a teaching tool in manual therapy professions curriculum [e.g., chiropractic (11) and physiotherapy (12)].

Force-time characteristics of SMT and MOB measured by different devices have been summarized in previous reviews (6, 13, 14). However, pooling measures recorded by different devices might not be appropriate. Specifically, it has been shown that forces with specific characteristics measured at the clinician-patient interface present different characteristics than the ones measured simultaneously at the patient-table interface (15). Additionally, even though different devices might measure force-time characteristics in a similar manner (i.e., at the same interface), each device has its specific metrologic properties (e.g., reliability, validity, specificity, and sensitivity), which influence the confidence in the measured values. Identifying and reporting on device characteristics in a comprehensive manner is therefore necessary to identify the most appropriate device for a specific purpose or the need to develop a new device.

Although metrologic properties are important factors to consider when determining which device to use for measuring SMT or MOB force-time characteristics, other factors might facilitate or limit the use of a specific device. These factors and their relative importance, such as the cost of the device, its ease of use and its versatility also vary and should be considered based on the context of use. To our knowledge, the factors potentially facilitating or limiting the use of devices measuring SMT/MOB force-time characteristics have not yet been thoroughly mapped. Conducting a scoping review of the devices used to measure SMT/MOB force-time characteristics should not only provide a comprehensive description and side-by-side comparison of the devices' metrologic properties, but should also provide the opportunity to delineate the facilitating and limiting factors discussed by the authors, which are important considerations when choosing a device The results of such a review could not only facilitate the development of future studies by providing a comprehensive description of devices' characteristics, but also identify gaps and areas of improvement that, in turn, could guide the development of new devices to be implemented in manual therapy profession curriculums and research programs.

The primary aim of this scoping review was therefore to characterize the metrologic properties of devices used to quantify force-time characteristics during SMT and MOB. The secondary aim was to determine which factors may facilitate or limit the use of those devices in terms of research, clinical application and education.

## Methods

A mixed-methods scoping review was selected as the most appropriate study design to achieve both quantitative (metrologic properties) and qualitative (facilitating and limiting factors) aims. The protocol was developed according to the Joanna Briggs Institute (JBI) convergent segregated approach to mixed-method reviews and included five key steps: (1) identifying the research question, (2) identifying the relevant studies, (3) identifying the inclusion and exclusion criteria, (4) charting the data, and (5) collating, summarizing and reporting results. The protocol was registered on OSF registry (DOI 10.17605/OSF.IO/UCXQZ).

### Identifying the Research Question

This mixed-methods scoping review was conducted to address the following questions: What are the metrologic properties of the devices used to quantify force-time characteristics during SMT and MOB and which factors potentially facilitate or limit their use within the context of research, education and clinical practice?

### Identifying Relevant Studies

The search strategy was developed with the assistance of a librarian with expertise in health sciences from the Université du Québec à Trois-Rivières. The searches in databases were conducted from inception to June 30, 2021. The following databases were searched: Index to Chiropractic literature (ICL), Cochrane Central Library, Cumulative Index to Nursing and Allied Health Literature (CINAHL) and Medline. The following terms (MESH or non-MESH) were searched in combination: [(device^*^ OR tool^*^ OR instrument^*^ OR manikin^*^ OR mannequin^*^ OR simulat^*^) AND (mobilization^*^ OR mobilization^*^ OR manipulation^*^ OR “manual therapy”) AND (spinal OR musculoskeletal OR osteopath^*^ OR physiotherap^*^ OR chiropract^*^ OR lumba^*^ OR back^*^ OR cervical^*^ OR thora^*^ OR neck)] AND [(acceptability OR appreciation OR evaluation OR view^*^ OR attitude^*^ OR opinion^*^ OR acceptance OR assessment OR “clinical application^*^” OR learning OR feedback OR “motor skill^*^” OR “motor learning” OR education OR research OR teaching^*^ OR training) OR (availability OR force^*^ OR fidelity OR accuracy OR consistency OR precision OR repeatability OR reproducibility OR metrology OR reliability OR validity OR price OR cost OR usability)]. The search strategy was first developed for Medline (see OSF registry) and subsequently adapted to the other databases. References from relevant studies and from identified reviews were manually searched. Results from databases searches were first imported into Endnote (EndNote X9.3.3, Clarivate™) to remove duplicates. References were then imported into the Covidence software to manage the reviews (Covidence systematic review software, Veritas Health Innovation, Melbourne, Australia).

### Inclusion and Exclusion Criteria

To be included, the studies had to: (1) be published in a peer-reviewed journal, (2) be written in either French or English, (3) have used, or present a device measuring SMT and/or MOB force-time characteristics on humans or with a potential use on humans, and (4) report any metrologic property of the device and/or discuss factors facilitating or limiting the use of devices within the context of research, education or clinical practice. Included studies had to present an observational, laboratory or experimental design. Technical or validation reports were also included. Reviews were initially included in case they identified additional facilitating or limiting factors. Studies were excluded if the modality was applied to non-spinal locations (i.e., upper or lower limbs or skull) or if it focused on other manual therapy techniques such as distraction, soft tissue manipulation or patient self-manipulation. Studies not conducted on humans were included if the device was intended for future use on humans (e.g., technical reports). The inclusion and exclusion criteria are detailed in Table 1.

**Table 1:** Inclusion and exclusion criteria.

**Category**	**Inclusion**	**Exclusion**
Language	•English or French	
Sample	•Device used to measure force time-characteristics	
Phenomenon of interest	•Spinal manipulation or mobilization applied to humans (or with a potential to be used on humans)	•Any other manual therapy modality (e.g., soft tissue technique) •Therapies not executed on the spine •Patient self-manipulation
Design	All peer-reviewed studies including: •Observational clinical studies •Laboratory studies •Randomized or non-randomized experimental studies •Technical or validation reports •Literature reviews (narrative, systematic, etc.)^*^	•Opinions, commentaries or editorials •Letters to the editor and editor responses •Case reports and case series studies •Conference proceedings •Theses •Book •Other non-peer reviewed publications
Evaluation	•Factors potentially facilitating or limiting the use of the device within the context of research, education or clinical practice •Metrologic properties of the devices (e.g., validity, reliability, reproducibility)	
Research type	•Quantitative and/or qualitative data	

* *Literature reviews were included if qualitative data not reported in original studies were identified*.

### Screening and Agreement

Following the removal of duplicates, a two-phase screening process was conducted. First, title and abstracts from all studies were screened by two independent reviewers (M-A.M. and P.R.) and classified as relevant, possibly relevant or irrelevant. Next, the full texts of relevant and possibly relevant studies were independently screened by the same two reviewers to identify the final studies to be included in this scoping review. If a study was excluded only by one reviewer during phase I or II, disagreement was resolved by discussion between the reviewers. If no consensus was reached, a third reviewer (M.D. or I.P.) was involved.

### Charting the Data

The following data were extracted from the included studies: authors and years of publication, objectives, study design, measurement interface (i.e., clinician-patient or patient-table), device description and whether it was the same device used in another study, metrologic properties, and factors facilitating/limiting the use of the device. All data were extracted using a standardized form by M.A.M. and reviewed by I.P. to minimize potential errors.

### Collating, Summarizing, and Reporting Results

A descriptive numerical analysis was first conducted to provide an overview of the results. Metrologic properties for each device were then classified by type, and data were extracted whenever available. Exact sentences regarding facilitating or limiting factors were extracted when available. One reviewer (M.A.M.) coded each sentence and defined thematic categories. Another reviewer (I.P.) initially reviewed the codes and themes. The two investigators met via teleconference to reach coding consensus and refine thematic categories. Facilitating and limiting factors were finally summarized based on themes and device interfaces (i.e., measuring at the clinician-patient interface or at the patient-table interface).

## Results

### Descriptive Numerical Analysis

**Figure 1:**
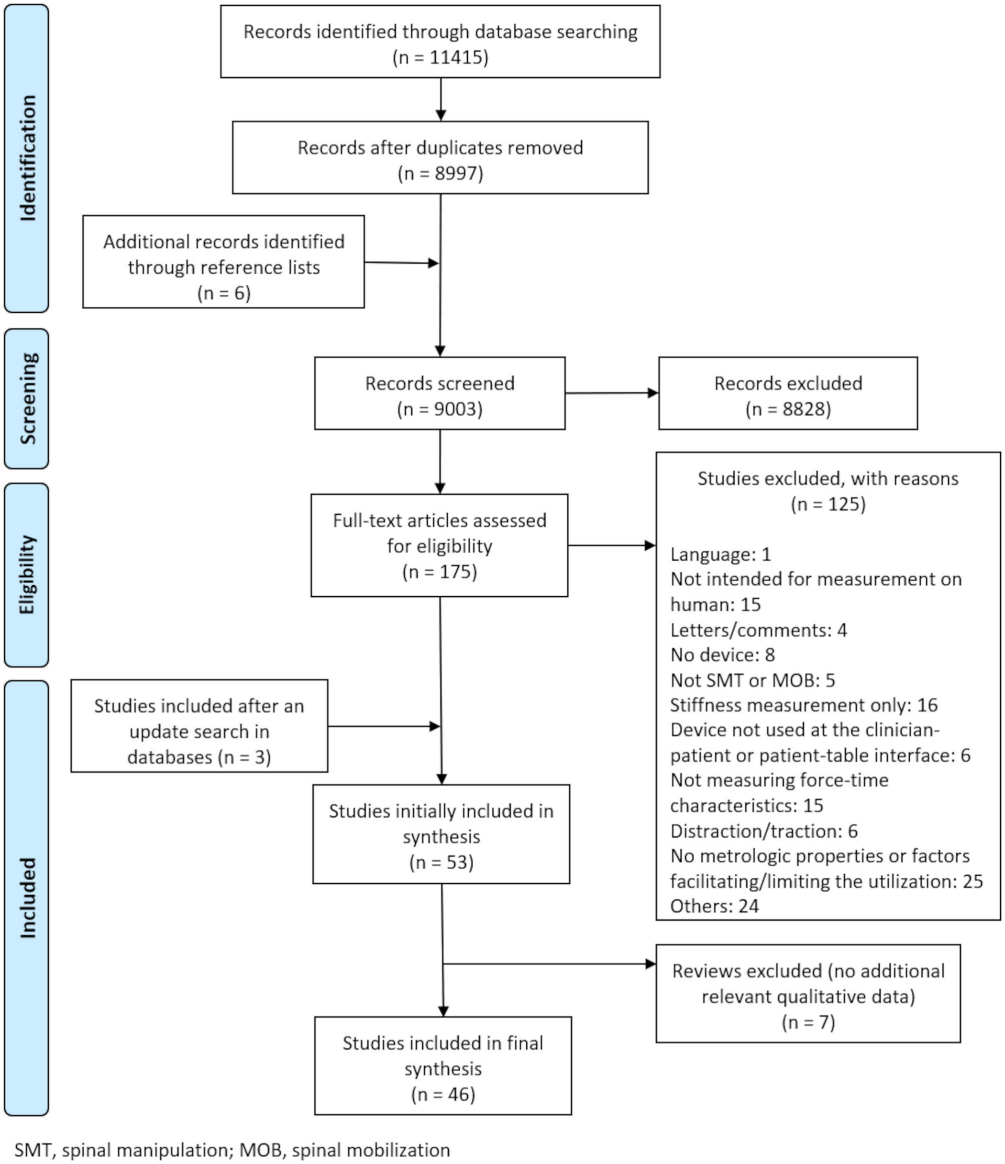
PRISMA diagram.

Figure 1 presents the PRISMA diagram of the current scoping review. The literature search was initially conducted from inception to June 19, 2020, and yielded 11,415 references, of which 8,997 were screened after removal of duplicates. Screening of the titles and abstracts resulted in the exclusion of 8,828 studies and an additional 132 studies were excluded after full-text screening. The reference list of the included studies was searched and yielded the identification of 6 additional original studies. All disagreements were resolved by consensus between the two reviewers. The literature search was updated on June 30, 2021, and led to the identification of three additional articles fulfilling the inclusion criteria. Forty-six studies were deemed relevant and were included in this review (7–12, 15–54). The literature search also revealed seven literature reviews from which no additional original studies were identified (2, 6, 55–59). No additional facilitating or limiting factors were identified from the initially included reviews and, therefore, all reviews were excluded.

**Figure 2:**
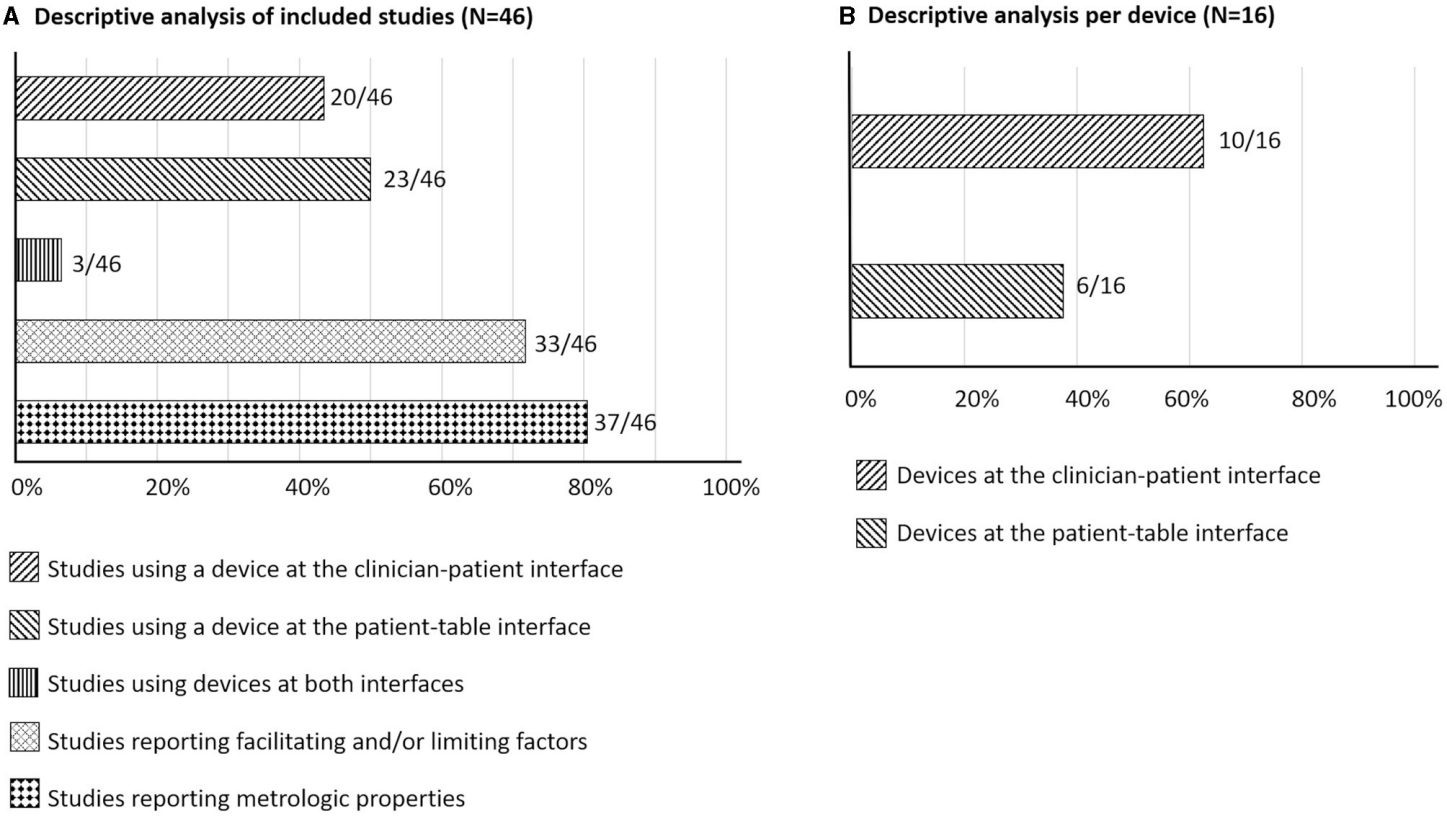
Descriptive numerical analysis **(A)** of included studies and **(B)** per device. Percentage of the total number of studies or devices are presented in the horizontal axis.

The characteristics of the included studies and both quantitative and qualitative data are reported in Supplementary Table 1 and visually presented in Figure 2. From the 46 included studies, 20 (43.5%) used a device to measure SMT or MOB force-time characteristics at the clinician-patient interface (7, 11, 12, 24, 26, 28–31, 37, 39, 40, 44, 46–52), 23 (50%) at the patient-table interface (9, 10, 15–23, 25, 27, 32–36, 38, 42, 45, 53, 54), and 3 (6.5%) at both interfaces (8, 41, 43). A total of 10 different devices measured SMT/MOB force-time characteristics at clinician-patient interfaces and 6 at patient-table interfaces. In light of the quantitative data, 37 (80.4%) studies reported at least one metrologic property (7–10, 12, 15–29, 31–34, 36–38, 40, 42–45, 47, 49, 50, 53, 54), with 16 (34.8%) reporting previously published results or referring to other studies (8, 12, 15, 22, 23, 27, 33, 34, 36, 38, 42, 43, 45, 50, 53, 54). On the other hand, facilitating and limiting factors were identified in 18 (39.1%) studies measuring forces at the clinician-patient interface (7, 11, 12, 24, 26, 28–31, 37, 39, 40, 44, 46–48, 51, 52), 13 (28.3%) studies measuring forces at the patient-table interface (10, 17, 19, 20, 22, 23, 25, 32–36, 38), and in 2 (4.3%) studies including measurement at both interfaces (8, 41).

### Metrologic Properties

Supplementary Table 2 presents the metrologic properties reported for each device as well as the modality evaluated and the targeted spine region. With exception of device *xvi*, at least one metrologic property was reported for each device. Eight properties were reported for device *xiii*, a standard mobilization couch adapted with load cells, four properties were reported for devices *ix* and *xi*, three for devices *v, viii*, and *xiv*, two for devices *ii, vi, x*, and *xv*, and one property was reported for devices *i, iii, iv, vii*, and *xii*.

**Figure 3:**
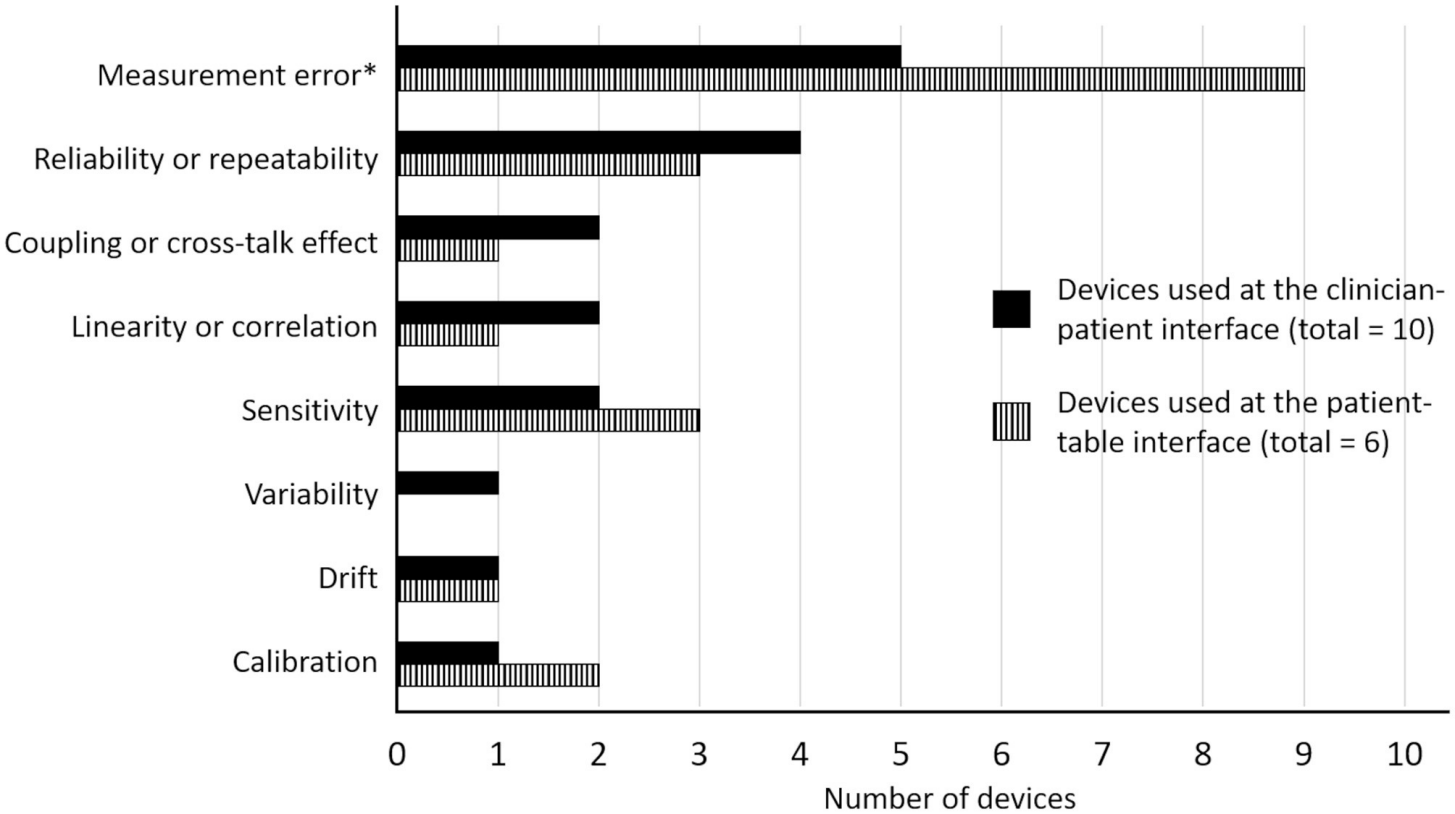
Reporting frequency of the metrologic properties for devices used at the clinician-patient interface and at the patient-table interface. * Measurement error was reported as the device validity, accuracy, fidelity or calibration.

All authors stated that their device was deemed appropriate for their purpose. The most common evaluated property was the calculation of the measurement error (raw or percentage) of a force measured simultaneously by the device and a gold standard, which was usually a load cell or force plate (devices *ii, iv, v, x*, and *xiv*). Measurement error was also assessed by comparing the force/pressure measured by the device while a known weight was directly placed on the device (devices *viii, ix, xi, xiv*, and *xv*). In both cases, authors referred either to the device accuracy (devices *ii, iv, viii, ix, xi, xiv* and *xv*), validity (devices *v*), fidelity (device *xiv*) or calibration (devices *x*). Accuracy was also reported for other devices, but how this property was determined was not described (devices *i, v, vi, vii, xii*, and *xiii*). The second most common property reported was the error between several measurements of the same event and referred to the device reliability (devices *x, xi, xv, ix*, and *xiii*) or repeatability (device *viii*). The device sensitivity was reported for four devices (device *ii*, vi, *ix*, and *xiii*). This property was defined as the minimal detectable signal over the noise inherent to the device for device *xiii* and as the smallest measurable output and output changes for device *ix*. Static sensitivity, the slope of the least square calibration regression line, was also reported for device *ix*. How the sensitivity of devices *ii* and *vi* was measured was not reported. Linearity, described as the correlation coefficient computed between different applied force/pressure and the measured value by the device, was reported for three devices (devices *iii, xi* and *xiii*). Two devices reported the signal drift, which was defined as the variation in the signal over time when a known load laid steadily on the sensor (device *ix* and *xiii*). Coupling or crosstalk effect was also reported, and was described as the percentage of the load measured in the caudad-cephalad and medial-lateral axes when a known load is applied vertically on the load cells (device *xi* and *xiii*). In line with the coupling effect, device *v*, a sensing polyester film, was reported to have some shear components when the force was applied perpendicular to the surface. Variability, that is the variation in the load measured by the device in response to changes in the point of load application, was reported for the mobilization couch adapted with load cells (device *xiii*). Finally, the calibration procedure was reported for three devices (devices *v, viii, and xiii*). Reporting frequency of each metrologic property can be visualized on Figure 3.

### Factors Facilitating Device Use

#### Metrological/Intrinsic Properties

Few studies commented on the importance of the biomechanical or technical aspects of devices used at the clinician-patient interface. Kirstukas and Backman (8) mentioned that using the polyester sensing film (device *v*) does not affect the biomechanical outcome of the technique. Kope et al. (44) reported that their applied maximal pressure did not affect the sensors, nor did it saturate the data acquisition system of their nail-affixed device (device *ii*). Their device also allowed to record the total range of MOB forces with small deflections on the nail. Lee et al. (29) reported that their water-filed pressure pad (device *iii*) was adequate to measure MOB forces usually applied to the cervical spine. Van Zoest et al. (30) highlighted that the use of a 3D contact force measurement system (device *ix*) not only measures the force magnitude, but also provides information on the direction of its application. Gudavalli et al. (39) mentioned that, based on their study results, a sampling frequency of 100 Hz provides reasonably accurate measurement of SMT force-time characteristics for students and clinicians training. The authors argue that the physical characteristics of the device, such as its pliability (quality of being easily bent), should be a more important criterion than the sampling frequency in this context. From a biomechanical perspective, Chiradejnant et al. (22) mentioned that their device (device *xi*) could provide more accurate data compared to the ones used in previous studies. Harms et al. (17) reported that the calibration of their instrumented couch (device *xiii*) showed suitable characteristics to measure SMT techniques. In a subsequent study (20), they mentioned that their couch was a valid tool to measure MOB forces applied to the lumbar spine, and that it is useful to record forces applied to a patient in a context of manual therapy techniques assessment.

Metrological/intrinsic properties were not reported as facilitating factors for devices at the patient-table interface (9, 10, 15–23, 25, 27, 32–36, 38, 42, 45, 53, 54).

#### User-Friendliness and Versatility

Devices utilized at the clinician-patient interface were commonly reported as small, thin, simple and easy to transport [devices *ii* (44), *iv* (40), *vi* (26), *vii* (12), and *ix* (30)]. These characteristics allow clinicians to perform their regular movements without interference from the device. Van Zoest et al. (30) mentioned that their hand palm-held device (device *ix*) can be used on different parts of the body, which makes it versatile. Similarly, Smit et al. (26) found the two force transducers device (device *vi*) to be compact, easy to set up, to transport and to store. The computer program was also reported to be uncomplicated. Moreover, due to the small size of the sensors, the placement of the device was reported to be easy. Tuttle and Jacuinde (37) mentioned that their pressure sensors (device *viii*) were easy to calibrate.

Howarth et al. (41) is one of the few studies that used a device at the clinician-patient interface (device *iv*) and at the patient-table interface (device *xiv*). They mentioned that their linked segment model requires a short time of utilization and is versatile, so different patient postures can be modeled and patient body characteristics can be taken into consideration.

Harms et al. (20) were the only authors to report about user-friendliness or versatility of a patient-table interface device. They mentioned that their instrumented couch (device *xiii*) is a versatile couch that can be used for many applications in the context of manual therapy.

#### Cost and Durability

Three devices used at the clinician-patient interface were reported as inexpensive or cost-effective [devices *ii* (44), *vi* (26), and *viii* (37)]. Specifically, Tuttle and Jacuinde (37) developed a low-cost sensor-based device (device *viii*) from parts available from electronic suppliers, which did not require specialist skills to construct (cost around $30 US dollars). Kope et al. (44) stated that their nail-affixed device (device *ii*) was cost-effective without mentioning its cost. Finally, the device described in Smit et al. (26) (device *vi*) was reported as being inexpensive, in addition to being made of two highly durable force transducers.

Cost and durability were not reported as facilitating factors for devices at the patient-table interface (9, 10, 15–23, 25, 27, 32–36, 38, 42, 45, 53, 54).

#### Technique Application

Some authors used or developed a device measuring forces at the clinician-patient interface and took into consideration the comfort of either the clinician or the patient [devices *ix* (28) and *x* (31, 47, 48)]. In Van Zoest and Gosselin (28), no discomfort was reported by the patient when the 3D contact force component measurement system (device *ix*) was used. The three studies by Waddington et al. (47, 48) aimed to design a device (device *x*) which would be comfortable for both the clinician and the patient as well as efficient. Waddington et al. (47) believed that their mobilizing dynamometer could decrease clinicians' risk of wrist and hand injury. In a subsequent study, in which they modified the device, they mentioned that their manual therapy dynamometer offered a good compromise between patient comfort and placement specificity (31). More specifically, they explained that the size of the rubber tip was greater than the contact area of the pisiform grip (i.e., SMT using the hypothenar region to contact the transverse process), but could still fit over a vertebra. In a third study, their modified dynamometer with a contoured soft rubber tip (Mobdyn IIb) was found to be acceptably comfortable for the clinician and the patient (48). The authors mentioned that the visual access to a dial improves clinician comfort by allowing them to control their force and, therefore, minimize it. Alongside comfort, similarity between performing with or without the device was also reported for some devices [devices *ii* (44), *vii* (12), *viii* (37), *ix* (28)]. Devices *vii* and *ix* did not affect the way clinicians perform SMT or MOB, and participants applying the therapy felt comfortable and confident using the device (12, 28). Devices *ii, viii* and *ix* were reported as not significantly affecting the applied technique (28, 37, 44). Petersen et al. (12) also mentioned that their load pad force monitoring device (device *vii*) minimized disturbance proprioception of the clinician.

Recurring comments for devices measuring at the patient-table interface are that this type of device allows the practitioners to perform SMT/MOB without having the measuring device between their hands and the patient's back [devices *xiii* (20) and *xv* (10, 32–36)]. Harms et al. (20) mentioned that their mobilization couch adapted with load cells (device *xiii*) constitutes the first device to allow the clinician to deliver the therapy as usual (without being affected by the measuring forces system). Devices measuring forces at the patient-table interface are therefore believed to allow SMT and MOB applications in a more clinically relevant manner, while contributing to the understanding of SMT/MOB biomechanics.

#### Feedback

Some devices used at the clinician-patient interface were reported as offering a good real-time feedback tool for students and clinicians [devices *ii* (44), *iv* (40), *vi* (26), *vii* (12), *viii* (37), and *ix* (24)]. It was stated that the load pad force monitoring device (device *vii*) would be useful in educational context to provide feedback to students and improve their learning of manual skills (12, 46). Tuttle and Jacuinde (37) pressure sensors (device *viii*) was also reported to be helpful for students in the modulation of applied forces. Gudavalli and Rowell (40) mentioned that their miniature three-dimensional force transducer device could eventually be used to measure biomechanical variables directly from patients in clinical context. Petersen et al. (12, 46) both mentioned that their load pad force monitoring device (device *vii*) offers the possibility to students to compare themselves with an expert standard, which can result in a more accurate application of force during their manual therapy learning. Petersen et al. (12) participants, first-year physiotherapy students, mentioned feeling confident using the device and that it would help them to learn and retain manual skills. Waddington et al. (48) stated that the immediate feedback provided by their modified manual therapy dynamometer (device *x*) would allow the clinician to apply a certain MOB force and stop when the desired force is reached. The same authors also claimed that the read-out of their device would allow eliminating variability while performing manual therapy. Finally, regarding Kope et al. (44) nail-affixed device (device *ii*), the authors mentioned that objective feedback could potentially help reduce adverse events associated with excessive forces in clinical practice as well as in educational contexts.

Feedback was also reported as a facilitating factor for devices at the patient-table interface [devices *xiv* (23) and *xv* (34, 35)]. Triano et al. (23) mentioned that the use of an instrumented table (device *xiv*) to measure biomechanical data (using an inverse dynamic method) is advantageous to inform on the total load passing through the patient. Snodgrass et al. (34, 35) mentioned that the use of an instrumented table (device *xv*) allows students to receive feedback about their MOB forces which could help them reproduce forces similar to the one of therapists in practice. They added that the knowledge of their own force application allowed by the instrumented table is the first step in establishing strategies in the learning of students.

### Factors Limiting Device Utilization

#### Metrological/Intrinsic Properties

Data acquisition frequency and load limits of some devices utilized at the clinician-patient interface have been recognized as limiting factors [devices *iii* (29), *iv* (39), *viii* (37)]. Gudavalli et al. (39) mentioned that other devices such as force sensors or force plates allow recording data at higher frequencies than their three-dimensional force transducer (device *iv*). It should be noted that the authors highlighted that higher data acquisition frequency may not result in a higher accuracy when measuring SMT force-time characteristics. On the other hand, Lee et al. (29) (device *iii*) and Smit et al. (26) (device *vii*) reported that their device is not accurate for load >70 N and >500 grams, respectively, which are loads commonly reached during SMTs. Other sensors should therefore be used when higher loads are expected. Tuttle and Jacuinde (37) also reported that their pressure sensors (device *viii*) is limited in terms of range of forces. In line with the metrologic properties, Kawchuk and Herzog (51) reported that their pressure mat (device *i*) cannot record all forces during rotational cervical manipulation, due to the non perpendicular component of the force applied to the device.

Metrological/intrinsic properties were also reported as limiting factors for devices at the patient-table interface [devices *xiv* (10) and *xv* (33–35)]. Snodgrass et al. (33–35) mentioned that their instrumented table (device *xv*) recorded global forces acting on the table instead of the force acting on a specific vertebra. The authors also highlighted that due to the placement of the sensor under the patient, the measured force is the one transmitted by the patient to the table and not directly the force applied by the clinician (10). Movements of the patient during the SMT or MOB could therefore alter the data (10). Likewise, Snodgrass et al. (32) (device *xv*) and Harms et al. (16) (device *xiii*) both reported that horizontal/shear forces are less accurately recorded or not recorded by their instrumented table than the force applied perpendicularly to their device.

#### User-Friendliness and Versatility

The same 3D contact force component measurement system (device *ix*) was used at the clinician-patient interface in three studies (24, 28, 30), and the recurring comment was that their device does not allow for small specific point of contact such as transverse and spinous processes. Kope et al. (44) also mentioned that patient and clinician positioning is limited due to the potential loss of force data during techniques generating forces that are not applied perpendicularly to their custom sensor worn on the nail (device *ii*), while Tuttle and Jacuinde (37) highlighted the possibility of data loss when the force is partly applied toward a body part outside their pressure sensor (device *viii*).

User-friendliness and versatility were not reported as limiting factors for devices at the patient-table interface (9, 10, 15–23, 25, 27, 32–36, 38, 42, 45, 53, 54). However, Waddington and Adams (31), who used a device at the clinician-patient interface (device *x*), reported that an instrumented plinth is a functional research tool, but that this type of device does not provide a practical solution to determining MOB forces applied by a therapist in a clinical setting.

#### Cost and Durability

Cost and durability were not reported by authors as limiting factors for devices utilized at the clinician-patient (7, 12, 24, 26, 28–31, 37, 39, 40, 44, 46–52, 60) or at the patient-table interfaces (9, 10, 15–23, 25, 27, 32–36, 38, 42, 45, 53, 54). However, Waddington and Adams (31) mentioned that instrumented plinth is quite an expensive solution in comparison to their device at the clinician-patient interface (device *x*).

#### Technique Application

The most discussed limiting factor of devices used at the clinician-patient interface was that such device creates an obstacle between the clinician and the patient's back, resulting in the alteration of the feeling of the patient tissue compliance by the clinician or of tactile feedback [devices *iv* (11, 40) and *ix* (24)], and potential alteration of the treatment application [device *ii* (44)]. Waddington et al. (47) also reported that the patient loses the comfortable sensation of the therapist's hand on the back because of their modified manual therapy dynamometer (device *x*). Using the same device (device *x*), Waddington et al. (47, 48) reported that both the clinician and patient comfort were decreased due to the hard rubber tip of the device when the clinician applied a MOB with the pisiform grip technique compared to the same technique delivered without the use of a device, and that their device frame caused clinician hands to be too elevated relatively to the patient's back. Herzog et al. (7) also mentioned that the use of any kind of pressure sensor between the clinician and the patient will inevitability have an influence on the real pressure distribution. Howarth et al. (41) reinforce this limitation by mentioning that, when using their miniature three-dimensional force transducer (device *iv*), clinician could not directly contact the patient to ensure that the totality of hand contact forces passed through the device. As a result, the hand contact adopted by the clinicians was slightly different than usual.

Technique application was not reported as a limiting factor for devices at the patient-table interface (9, 10, 15–23, 25, 27, 32–36, 38, 42, 45, 53, 54).

#### Feedback

Among devices at the clinician-patient interface, only Tuttle and Jacuinde (37) reported feedback as a limiting factor of their device (device *viii*). Indeed, the authors stated that their pressure sensors do not provide feedback as accurately as other devices.

Feedback was not reported as a limiting factor for devices at the patient-table interface (9, 10, 15–23, 25, 27, 32–36, 38, 42, 45, 53, 54).

## Discussion

The high use of SMT and MOB by manual therapists (e.g., chiropractors, physiotherapists, osteopaths) worldwide to treat musculoskeletal pain made these interventions the focus of several clinical, educational and mechanistic investigations. With the increase in knowledge on the potential role of the SMT/MOB force-time characteristics on the mechanisms underlying the clinical effect of these manual therapy modalities, a growing number of studies measuring SMT or MOB force-time characteristics has also been observed. This mixed-methods scoping review examined the literature to synthesize the metrologic properties of the available devices to measure SMT/MOB force-time characteristics and identified potential factors facilitating or limiting their use within the context of research, education or clinical practice. Our findings revealed that, to date, numerous devices have been developed and often improved over time for measuring manual therapy force characteristics. Although at least one metrologic property was reported for all devices except for one, a consensus on standardized terminology is lacking. The qualitative data revealed that there has been very little empirical exploration of the factors potentially facilitating or limiting the use of these devices with most factors only based on the opinion of the authors.

### Available Devices

Although previous literature reviews synthesized studies using devices to measure manual therapy force-time characteristics such as total peak force or rate of force application, the main interest of these reviews were the results relevant to manual therapy biomechanics or learning (2, 6, 55–59). The current review is therefore the first to report the metrologic properties and the factors facilitating and limiting the use of the devices that have been previously used in the literature related to manual therapy. A focus was placed on devices developed or adapted to measure SMT or MOB force-time characteristics delivered on human spine. Devices were categorized into those measuring the force/pressure/load directly at the site of application (clinician-patient interface) and those measuring indirectly through the reaction forces of the patient's body on the sensor (patient-table interface). Interestingly, almost the double number of devices measuring at the clinician-patient interface (*n* = 10) than at the patient-table interface (*n* = 6) were identified. This may reflect not only researchers' creativity in adapting commercially available devices to measure SMT and MOB, but also the variety and availability of such devices. In contrast to devices at the clinician-patient interface that were first the subject of publication in the early 1990s (49), the first publications including a device at the patient-table interface were published in 1995 (16, 17). However, 57% (26/46) publications included a device at the patient-table interface (23/46 or 50% for the clinician-patient interfaces) highlighting the higher number of publications using the same device at the patient-table interface. Indeed, one of the instrumented tables with force plate (device *xiv*) was reported in 11 studies (15, 19, 23, 25, 27, 41–43, 45, 53, 54). Future investigations should focus on the identification of the most appropriate technology depending on the assessment context. This could be achieved through expert consensus and could lead to the development of a theoretical framework for future development on this topic.

### Metrologic Properties

Metrologic properties include different concepts that are often misused or used interchangeably within the literature (61). In the current review, as many as four different terms were used for the description of the same measurement. Specifically, accuracy, validity, fidelity, and calibration were all defined as the measurement error between either the evaluated device and a gold standard device, or with a known weight lying on the device. An attempt was made to regroup metrologic properties evaluated in a similar manner so that the data could be synthesized comprehensively. However, how these properties were measured was not always defined (devices *i, vi, xii*, and *xiii* for their accuracy; devices *ii* and *vi* for their sensitivity) and few authors only refer to a study evaluating the device in a context not related to SMT or spinal MOB (devices *v* and *vii* for their accuracy). For this reason, it was not possible to contrast metrologic properties between devices. It is therefore crucial to define a common and standardized terminology to be consistently used in manual therapy studies. It is also fundamental that future studies properly describe how each metrologic property is measured.

Squara et al. (62, 63) recently published a framework based on the International Bureau of Weights and Measures (64), but adapted to perioperative and intensive care medicine. This framework defines the terms related to quantities and units, properties of measurements, devices for measurement, properties of measuring devices, and measurement standards. Considering that Squara et al. (62, 63) framework is adapted to the medical field, it seems reasonable to use it as a reference point for a common and standardized terminology in the manual therapy field.

**Accuracy** is defined by Squara et al. (62, 63) as the closeness of agreement between a single value measured by the device and the real value, and is usually expressed as an error of measurement. A distinction between accuracy and trueness should also be done. **Trueness** is determined by the systematic measurement error or difference between the averaged measured value and the reference value obtained when several measurements are compared to the true value (e.g., several measurements of a known weight laid on the device). In the current study, the error of measurement was also associated with the terms validity, fidelity, and calibration, but no study used the term trueness, which, based on the measurement descriptions, would have been the most appropriate term. Interestingly, Waddington et al. (47) reported that the force measured by their modified dynamometer never exceeded 1 N of the real force, and referred to this assessment as their device calibration. However, this assessment would better reflect the device trueness. By definition, **calibration** is the comparison between the measure obtained by the device to a known reference standard. In case of improper calibration, a procedure of adjustment can be then performed.

**Precision** is another metrologic property, which can be expressed as variability (%) or a random measurement error. This property refers to the variability of replicate measurements of a same procedure without reference to the reference value, and includes both **repeatability** (several measurements of the same procedure done in a short period of time in the same exact condition) and **reproducibility** (several measurements of the same procedure done in variable conditions). In the studies included in the current review, authors seem to have used the term reliability instead of repeatability or reproducibility. It is worth noting that Harms et al. (20) assessed the variation in the load measured by their mobilization couch adapted with load cells (device *xiii*) in response to changes in the point of load application and referred to it as the device variability, but, based on Squara et al. (62), the term reproducibility would have been more appropriate.

**Sensitivity** is defined by Squara et al. (62) as the quotient of the change in an indication and the corresponding change in the quantity intended to be measured. In other words, sensitivity corresponds to the change in the load measured by the device in comparison with the real load variation. In the studies included in this review, sensitivity was defined as the smallest measurement output change (device *xiv*) or as the minimal signal detectable over the noise inherent in the system (device *xiii*). However, in both cases the measurements more appropriately reflect the device **resolution**, which corresponds to the smallest change in the measure that can be detected by the device. It is important to note that sensitivity can be measured throughout the measurement interval relevant for the context. How sensitivity varies throughout this interval refers to the **linearity**, which is, in fact, a mathematical property and not a metrologic one. Linearity was reported for three devices (*iii, xi*, and *xiii*) identified in this review, which all showed almost perfect linearity (*r* ≈ 0.99).

The measure of the device **drift**, reported for devices *ix* and *xiii*, corresponds with the definition provided by Squara et al. (62), i.e., the change over time of the value measured by the device when the value remains the same in reality. A device with no drift has an excellent or perfect **stability**. **Coupling or crosstalk** effect is not reported as a metrological property in Squara et al. (62, 63). However, this property is deemed important in the context of manual therapy, since part of a vertically applied load can be falsely measured on the cephalad-caudal and medial-lateral axes, as reported for devices *ix* and *xiii*. Finally, it is noteworthy to mention that **validity** is not considered a metrologic property, but a research construct. To determine the validity of a device, the relevant metrologic properties for the specific purpose have to be considered as a whole (62, 63). Specific instructions on how to measure each metrological property, with examples, are described in Squara et al. (62, 63).

### Facilitating and Limiting Factors

Potential factors facilitating and limiting the use of the devices included in this scoping review were collected qualitatively. With the exception of “comfort,” all other factors were subjective comments made from authors' experiences rather than an objective assessment. Technology acceptance models have been developed to predict technology usage by assessing factors that influence its acceptance (65, 66). While these models are often focused on the technology itself, two models have been used in health care to assess acceptance of health care information systems and technology: the Technology Acceptance Model (TAM) and the Unified Theory of Acceptance and Use of Technology (UTAUT) (67). While both models include the assessment of the t perceived usefulness and ease of use of the technology, UTAUT also includes social influence and facilitating conditions.

Perceived usefulness corresponds to the user's perception that using the technology will increase the task performance, and aligns with the metrologic/intrinsic properties and feedback themes identified in this study. Specifically, the metrological/intrinsic properties theme included comments related to the importance of the technical characteristics of the devices, and how these enable or restrict the proper measurement of manual therapy forces. The feedback theme emphasized the potential application of manual therapy real-time feedback in different areas, such as in education context.

Perceived ease of use corresponds to the user's perception that using the technology will be easy and effort-free. This aligns with the user-friendliness and versatility, as well as with technique application themes identified in this study. While the user-friendliness and versatility theme highlighted the characteristics that contributed to the device's ease of use (e.g., size, not interfering with movement, easy to transport, set up and store), technique application theme referred to the level of comfort of both clinicians and patients experienced while using the device to measure manual therapy forces and to the perceived realism of technique application by the clinician.

The social influence variable described in the UTAUT's model corresponds to the users' perception of others' believing that it is important to use this technology. This was not captured in any of the themes in our study, suggesting that social influence might not play a significant role when researchers are choosing the technology to use to measure manual therapy forces.

Facilitating conditions (from the UTAUT's model) corresponds to the organizational and infrastructure support to use the technology, and aligns with the cost and durability theme from this study. Costs and cost-benefit assessment (either objective or subjective) are most often considered when selecting the device, which was emphasized by the authors of studies included in this review.

The alignment between variables from technology acceptance models and the themes identified in this study indicates that while such models can indicate specific aspects that are important for a device's acceptance and usage to measure SMT and MOB forces, a specific model for this specific field might assist manufacturers to tailor their device's design to measure forces during manual therapy. This would also allow for a formal evaluation of the acceptance and use of force sensing devices in the context of manual therapy, which would inform the selection of the device to be used in future studies, and the design of future force sensing devices.

### Research, Education, and Clinical Implications of the Results

The results of this scoping review should inform researchers when designing future studies requiring the measurement of SMT or MOB force-time characteristics. Whenever possible, the use of an available device should be prioritized over developing a new one. This would allow resource-use efficiency, as well as measurement standardization across studies which, in turn, would facilitate combining data from different studies, and knowledge sharing.

Augmented feedback is considered a key feature of motor learning of any task, and manual therapy makes no exception (14). The quantity and type of feedback depends on several factors, including the motor task itself, the learner expertise, and the learning objective (68). When learning SMT and MOB, students often practice on their peers, following the observation of an expert (a clinician or a professor) performing the task. The expert then provides a subjective feedback to the student regarding their SMT or MOB execution (14). The inclusion of devices measuring SMT/MOB force-time characteristics into manual therapy professions curriculum offers the opportunity to provide objective feedback to the students. Early studies support the use of such devices to help students becoming more accurate and consistent during SMT or MOB delivery (12, 37, 46). In addition, students have been considering that the use of devices providing objective feedback helps them learn and retain manual skills (12, 69). Postgraduate programs, such as physiotherapy, chiropractic, naprapathy and osteopathy, looking to implement devices providing real-time quantitative feedback into their curriculum should consider the data presented in the current study.

Mechanisms underlying the clinical effects of SMT and MOB have not been entirely clarified, but researchers agree that they include both spinal and supraspinal events, as well as contextual factors (3, 70, 71). Given clinical practice recommendations to use conservative interventions, including SMT and MOB, to manage acute and chronic musculoskeletal pain, implementing tools measuring SMT/MOB force-time characteristics into clinical practice could help clinicians to adapt their treatment to each patient's individual biomechanics and needs. Safety could also be potentially enhanced by insuring the delivery of SMTs causing lower constraints on both the clinician's and patient's joints (44, 47). The use of devices into clinical practice remains to be further investigated.

### Strengths and Limitations

The main strength of this scoping review is the use of a mixed-methods involving both quantitative and qualitative data. It is worth noting that the facilitating and limiting factors identified in this scoping review were mostly based on the subjective opinion of the authors of the included studies. This, the relative importance of these factors in the use of the devices in research, educational or clinical setting could not be determined. Other factors might also not have been identified. Finally, due to the nature of this study and the lack of study comparing different devices, it was not possible to identify a specific device that should be used over the others.

## Conclusion

This mixed-methods scoping review mapped the devices used to measure manual therapy force-time characteristics and their metrologic properties. A total of 10 individual devices measuring at the clinician-patient interface and 6 devices measuring at the patient-table interface were identified. Although all studies, except for one, refer to metrologic properties, a consensus on terminology and appropriate standardization are lacking. Factors reported by authors as facilitating or limiting the use of their device in research, education or clinical practice were charted into five themes: user-friendliness and versatility, metrologic/intrinsic properties, cost and durability, technique application, and feedback. The results of this review could guide the choice of the device to use depending on the context and the facilitating and limiting factors to their utilization.

## Author Contributions

M-AM and IP contributed to study design, literature search design and studies review, data extraction and charting, and wrote the preliminary version of the manuscript. PR contributed to the literature search and studies review, data extraction, and critically reviewed the final manuscript. MF contributed to study design, literature search review, and editing of the preliminary version of the manuscript. MD contributed to study design, literature search and studies review, overall supervision of the project, and critically review the final manuscript. All authors contributed to the article and approved the submitted version.

## Funding

Funding for this study was provided by the Chaire de recherche internationale en santé neuromusculosquelettique and its partner, the Fondation chiropratique du Québec. The funding sources had no role in study design, in the collection, analysis and interpretation of data, in the writing of the report and in the decision to submit the article for publication.

## Conflict of Interest

The authors declare that the research was conducted in the absence of any commercial or financial relationships that could be construed as a potential conflict of interest.

## Publisher's Note

All claims expressed in this article are solely those of the authors and do not necessarily represent those of their affiliated organizations, or those of the publisher, the editors and the reviewers. Any product that may be evaluated in this article, or claim that may be made by its manufacturer, is not guaranteed or endorsed by the publisher.

## Abbreviations

SMT: spinal manipulation
MOB: spinal mobilization
ICL: Index to Chiropractic literature
CINAHL: Cumulative Index to Nursing and Allied Health Literature
TAM: Technology Acceptance Model
UTAUT: Unified Theory of Acceptance and Use of Technology.
