# Supplementary material for: Devices Used to Measure Force-Time Characteristics of Spinal Manipulations and Mobilizations: A Mixed-Methods Scoping Review on Metrologic Properties and Factors Influencing Use
Source: Front Pain Res (Lausanne). 2021 Oct 29;2:755877. doi: 10.3389/fpain.2021.755877 (PMC8915691; doi:10.3389/fpain.2021.755877)
Supplement: Supplementary file 1 [file Table_1.docx]

Supplementary Table 1. Extraction table of the 47 included original studies.

| Author  Year | Objective(s) | Study design | Interface measured and description of the device | Quantitative data | Qualitative data | |
| --- | --- | --- | --- | --- | --- | --- |
|  |  |  |  | Metrologic properties | Facilitating factors | Limiting factors |
| Cambridge et al. (60) | This paper examines three variations of a thoracic spine HVLA procedure to determine whether they are effectively different in force-time profiles. | Cross-sectional descriptive study | **Patient-table interface:** same as in Triano et al., (23). | Previously validated for fidelity of measures (25). | NA | NA |
| Chiradejnant et al. (22) | To describe the forces used when a central PA mobilization was applied to the lumbar spine in subjects with LBP and to predict the treatment force characteristics on the basis of physical therapist and patient characteristics and treatment details. | Cross-sectional descriptive study | **Patient-table interface:** same as in Chiradejnant et al. (8). | The preliminary study (8) demonstrated that the couch is highly reliable (ICC [2,1], 99% CI = 0.99-1.0) and accurate with an error of less than 2% obtained when measuring the application of force in all 3 axes. A linear relationship existed between the force readings and known weights (r^2^ = 0.9999). The cross-talk effect was less than 2%. | ¨It can be concluded that the couch may provide more accurate data about treatment forces than equipment used in previous studies. ¨ | NA |
| Chiradejnant et al. (8) | To develop a portable instrumented treatment couch that could be used to measure the forces that physiotherapists applied to their patients in three directions, and to evaluate the accuracy and reliability of the instrumented couch. | Validation and technical study | **Patient-table interface:** 7 load cells (XTRAN Pty, Ltd, Australia) mounted between the frame and the top of a standard treatment couch (Australia Pty, Ltd, Model Special, Victoria, Australia). The load cells were connected to an amplifier mounted on the couch frame. Four, two and one load cells were positioned in the vertical, medial-lateral and caudad-cephalad directions respectively. | **Accuracy (with a human laid on):** The maximum error is less than 2% and on average is less than 1% for measurements in all three directions.  **Test-retest reliability:** The couch produced highly reliable measurements of force in all three directions (99% CI = 0.99-1.00).  **Average coupling-effect**: When weights ranging from 12 to 970N is applied to the vertical direction, the average coupling-effect is 1.26% (range, 0.40-1.97%) in the medial-lateral direction and 0.68% (range, 0.02-1.54%) in the caudad-cephalad direction. | NA | NA |
| Conradie and Wilsdorf (57) | To determine whether experienced physiotherapists apply equal magnitude of force during a grade I central PA mobilisation technique on the cervical spine. An additional aim was to determine the variation in the magnitude of force applied by each individual physiotherapist between two repetitions of the same technique. | Cross-sectional descriptive study | **Clinician-patient interface:** Same as Smit et al. (26). | NA | NA | ¨The physiotherapist’s feedback about the influence of the sensors on the technique indicated that only four subjects (25%) reported a slight influence on the tactile feedback, without necessarily affecting the magnitude of force applied during a grade I central PA mobilization technique.¨ |
| Conway et al. (55) | To measure the forces exerted during SMT in the thoracic spine simultaneously with corresponding cavitation signals. | Cross-sectional descriptive study | **Clinician-patient interface**: Same as Hessell et al. (54). | The accuracy of the force values obtained during SMT were estimated to be within 7% based on calibration experiments performed previously (54). | NA | NA |
| Goodsell et al. (21) | To establish the short-term effects of lumbar posteroanterior mobilization in patients with low-back pain, compared with a control intervention. | Cross-over experimental study | **Patient-table interface:** Custom-made instrumented treatment couch that quantified the vertical component of forces. The couch comprises a steel-framed treatment bed, 4 compression load cells (Precision Transducer Group, LPX-50, Precision Transducers, Castle Hill, New South Wales, Australia) interposed between the legs of the treatment couch and the floor, a summing amplifier, and a chart recorder. | Acceptable accuracy in recording known forces applied in a variety of locations on the couch surface. The maximum measurement error was 2.7%, equating to an error of 7N for loads of 270N. | NA | NA |
| Gudavalli and Rowell (40) | To report on the three-dimensional forces at the hand contact between the chiropractor and the simulated patient (asymptomatic volunteers) during side posture lumbar high velocity low amplitude spinal manipulations. | Pilot study | **Clinician-patient interface:** Device reported in Gudavalli et al. (39) placed between the doctor’s hand and the participant’s back. A thin layer of rubber padding was used between the transducer and the participant’s back, and the transducer was attached to the chiropractor’s glove using Velcro® brand fasteners. | The transducer was tested to compare the accuracy of force measurements in normal direction as well as shear direction with a Bertec force plate and found to have good agreement (less than 3% differences) between both sensors. | ¨The force measuring system developed and used in this study consisted of a laptop computer and was easily portable. ¨  ¨This system has the potential for taking it to any field clinicians’ office to gather data and give immediate feedback on the forces delivered. ¨  ¨The transducer is a two centimeter thick metal device with a rubber pad for the comfort of the participant. ¨ | ¨Tactile feedback is lost because of the transducer between the chiropractor’s hand and patients back.¨ |
| Gudavalli (10) | To determine the instantaneous rate of loading during manual HVLA SM in the lumbar and thoracic regions and compare to the average rates of loading. | Cross-sectional descriptive study | **Clinician-patient interface:** Device reported in Gudavalli et al. (39) placed between the doctor’s hand and the participant’s back. | NA | NA | ¨HVLA manipulations were delivered through a force transducer that is rigid with minimal cushion using a hard rubber case. The doctor may not feel the tissue compliance because of the transducer between the doctor’s hand and the subject’s back. ¨ |
| Gudavalli et al. (39) | To determine the minimal sampling rate required to accurately quantify the force-time characteristics of HVLA-SM, including preload and peak forces, duration of loading, duration of unloading, total duration, and rates of loading and unloading and to quantify the differences in those characteristics when data were collected at different sampling rates, whereas HVLA SM–type thrusts were repeatedly delivered to a force transducer by experienced doctors of chiropractic (DCs). | Cross-sectional descriptive study | **Clinician-patient interface:** Miniature force transducer (Model no. mini 45; ATI Industrial Automation, Apex, NC) placed on the top of a force plate (Model no. 4060-NC; Bertec, Inc, Columbus, OH). The transducer has a diameter of 45mm, a thickness of 15.7mm, a contact surface of 15.9cm^2^ and is capable of measuring 3-dimensional forces and moments. | NA | ¨Some equipment such as thin film sensors allows sampling rates in the order of only 50 to 100 Hz. Based on the findings of this study, this equipment may be used without loss of accuracy. This will allow researchers the confidence to apply these sensors with lower sampling rate in their studies, while taking advantage of the pliable nature of these devices that make them more suitable for measurement between doctor's hand and the participant's spine.¨  ¨Smaller sampling frequencies give reasonably accurate results can be useful to select the equipment for measuring forces and design future studies. This implies that training of students and clinicians can be done using low sampling rate force-measuring equipment that is more pliable and, therefore, causes less interference with their normal delivery of the thrust. ¨ | ¨Other equipment such as force sensors and force plates has the capacity to sample at much higher frequencies. Higher frequencies may be needed for some applications. However, in some applications such as monitoring force-time profiles of HVLA-SM, sampling at higher frequencies may not provide better accuracy and yet would produce excessive volume of data and consequently increase the data processing time and cost. ¨ |
| Harms et al. (19) | To define the characteristics of a typical mobilization force used on an asymptomatic lumbar spine in two subject groups of different age ranges. | Cross-sectional descriptive study | **Patient-table interface:** same as in Harms et al. (16). | Linearity was found to be high (0.99), and with sustained loading of forces equating to body weight, minimal drift was observed over a 10 min period (< 0.07%). Cross effects, expressed as a percentage of the signal in the primary axis, were generally < 2%. The variability of signal in response to changes in the point of load application was considered to be within acceptable limits of 2%.  The sensitivity of the couch was found to be better than 1N along the Z-axis, and 2 N along X- and Y-axes, well within the requirements necessary to record the smallest peak mobilization forces. | ¨The instrumented couch has been shown to provide a valuable method of measuring the mobilization forces used during treatment of the lumbar spine. This is the first system reported where the modus operandi of the therapist has not been affected by the measuring system. Its successful use in the clinical situation is an important advance with considerable potential in both research and teaching applications.¨  ¨The instrumented couch promises to be a versatile tool, with many applications in the field of manual therapy, an important advance in an area where the use of these techniques and their efficacy remain controversial. ¨ | NA |
| Harms and Bader (17) | To evaluate both the repeatability and reproducibility for one therapist, and to establish the variation in the characteristics of the mobilization force performed by a range of experienced therapists. | Cross-sectional descriptive study | **Patient-table interface:** same as in Harms et al. (16). | Reliable and sensitive over the range of forces applied during mobilization. | NA | NA |
| Harms et al. (15) | To document the first stages in the development of an instrumented mobilisation couch to allow measurement of the forces applied during spinal mobilisation. | Validation and technical study | **Patient-table interface:** device reported in Harms et al. (16) but modified with 4 load cells instead of 6. | Over 30 kg in the Z plane and 10 kg in X and Y planes, the system provided a linear response with a very small error component (r = 0.99) and a coefficient of variation of less than 1% in loads over 1 kg.  The sensitivity was determined by calculating the minimal signal detectable over the noise inherent in the system. This was found to be 20g in the vertical plane, 41g in the longitudinal plane and 74g in horizontal plane.  The load cell response was dependent on the point of force application. There is some shear load that is not being measured. | NA | ¨The load cell response was dependent on the point of force application. There is some shear load that is not being measured¨. |
| Harms et al. (16) | To describe the principles of the instrumentation of a mobilization couch suitable for measuring the forces applied during treatment. | Validation and technical study | **Patient-table interface:** A standard mobilization couch (Akron Therapy Products Ltd, UK) was modified to allow the couch top to be removed from the tubular steel frame. Transducers were mounted between the base and the couch top. The couch top was mounted on six bending-beam load cells (Model No: SHBxM, Revere Transducers Europe, The Netherlands). Three load cells were positioned in a tripod arrangement with vertical primary sensing axes. 'The three remaining load cells were mounted with horizontal-sensing axes in an arrangement that allowed calculation of forces directed along s and -y-axes. | **Accuracy:** Each bi-directional cell had a nominal capacity of 50 kg, although the manufacturers specified that each would be accurate 150% of this capacity. With sustained loading of forces equating to body weight, drift was found to be negligible over a 10-min period (<0.07%).  **Linearity:** Pearson product moment correlation (r) of 0,999 for the three axes. Coefficient of determination (r^2^) of 0,997 for x, and 0,999 for y and z.  **Variability:** within acceptable limits of 2%. | ¨Calibration procedures have demonstrated that the system has characteristics that are suitable for the measurement of spinal manipulative techniques.¨  ¨The design has potential for use in many other situations where the force delivered to a subject needs quantification, and is particularly appropriate for use in the assessment of manual therapy techniques.¨ | NA |
| Herzog et al. (7) | To quantify local measures of loading applied by the clinician on the volunteers during spinal manipulative treatments and to compare these local measures of loading with previously described global measures. | Cross-sectional descriptive study | **Clinician-patient interface:** Same as Hessell et al. (54). | The pressure mat has a stiffness similar to that of a bony prominence in the back covered by muscle, fat, and skin; therefore, the measurement errors in this study should be small (under 10%). | NA | ¨A word of caution about the force and pressure measurements using any kind of pressure sensor: the introduction of such a sensor will influence the “real” pressure distribution.¨ |
| Hessell et al. (54) | To develop an experimental procedure for determining the forces exerted by a chiropractor on the low back of patients with sacroiliac joint problems during spinal manipulative therapy, and to use this procedure to quantify these forces in a clinical situation. | Cross-sectional descriptive study | **Clinician-patient interface**: Thin (2-mm) flexible sensing pad (Emed Corporation, Munich, West Germany). | The accuracy of the pressure pad in measuring forces was determined to be within ± 7% of the actual value. | NA | NA |
| Howarth et al. (41) | To present the experimental setup, the development, and implementation of a new scalable model capable of efficiently handling data required to determine low back kinetics during high-velocity low-amplitude spinal manipulation (HVLA-SM). | Technical study | **Patient-table interface:** same as in Triano et al, (23).  **Clinician-patient interface:** The device reported in Gudavalli et al. (39) was used to measure applied forces and moments at the two contact locations between the clinician and participant’s thorax. One load cell was positioned over the participant’s shoulder for the clinician’s left-hand contact, and the second was positioned over the L5 spinous process for the clinician’s right hand/finger contact. | NA | ¨The advantages of this model over prior attempts are the brevity of experimental time (less than 1-hour), facility to model differing patient postures and applied loads as well as the built-in scalability to accommodate different patient body characteristics. ¨ | ¨The hand contacts adopted by the clinician in our investigation were similar, but not the same as those used by practicing clinicians. This was due to the fact that the clinician’s hands were not allowed to directly contact the patient to ensure that all hand contact force passed directly through the load cell that was interfaced with the clinician’s hand.¨ |
| Kawchuk and Herzog (56) | To observe chiropractors and their patients in a clinical situation and then determine whether five commonly used manipulative techniques possess unique biomechanical characteristics (fingerprints). | Cross-sectional descriptive study | **Clincian-patient interface:** Same as Hessell et al. (54). | NA | NA | “The pressure mat only registers forces applied perpendicular to its surface, yet the ROT procedure has a resultant force with a large component of force parallel to the surface of the pressure mat. therefore, only part of the actual force delivered during SMT using the ROT technique was recorded.” |
| Kirstukas and Backman (20) | To measure the time-varying normal contact pressure distribution at the physician–patient contact region and the magnitude of the time-varying resultant force vector at the supporting patient–table interface while chiropractic physicians performed the prone reinforced unilateral pisiform manipulation on symptom-free human subjects. | Cross-sectionnal descriptive study | **Clinician-patient interface:** 0.18-mm thick laminated polyester film that contains 1936 sensing regions in an 83.8-mm square area (Tekscan,Inc., South Boston, Mass).  **Patient-table interface:** A manipulation table outfitted with two six-component 1000-lb load cells (Model OR6-5, Advanced Mechanical Technology, Inc, Watertown, Mass). | **Clinician-patient interface:** Validation of the polyester film was demonstrated for two manual thrusts performed directly on the film overlaid on the load cell. For both thrusts the average absolute difference between the contact and support force magnitudes was 20N, less than 3% of peak force magnitude. The absolute difference between the peak contact and peak support force magnitudes for the two thrusts was 29N and 46N, or 4% and 6% of the respective peak force magnitudes.  The accuracy of the Tekscan system has been reported in the literature (e.g. Rose et al. 1992).  We used Tekscan’s bladder calibration device and the associated software package to equilibrate and calibrate the sensors over a range of values likely to be experienced during the experiment. We equilibrated the sensor at 690 kPa (100 psi) to ensure that every sensing element responded similarly to high pressures. We then performed a two-point calibration protocol with 345 and 690 kPa (50 and 100 psi) chosen as the two known pressures. | ¨We do not believe that the use of the contact sensor dramatically altered biomechanical outcomes. ¨ | NA |
| Kope et al. (44) | To implement a novel device to measure forces applied through the fingers and thumbs using a custom sensor that is worn on the finger nail much like an esthetic false nail. | Validation study | **Clinician-patient interface:** Novel device to measure forces applied through the finger and thumbs using a custom sensor that is worn on the finger nail much like an esthetic false nail. Each finger sensor was constructed from one uni axial foil type 350 Ω strain gauge (model CEA-13-062UW-350, Micro-Measurements Inc., Wendell, NC), which was cemented to a standard esthetic acrylic nail substrate (Nailene, Pacific World Corp., Aliso Viejo, CA). This device was mounted on the nail with its measuring axis oriented transversely across the finger. | In comparison to the load cell, postcalibrated testing demonstrated that sensor accuracy was 0.17 ± 0.02N, with a minimum sensitivity of 0.02N. | ¨The forces that are experienced at the finger pad cause small deflections in the therapist’s bone and soft tissues underlying the nail, which result in small deflections of the nail that are measured by the high-sensitivity sensor and converted into real-time force output. The low-profile sensor adds no more than 1.5mm thickness to the nail, allowing the user to move normally. the finger pad is not obstructed, which allows for the native tactile sensation to be experienced without interference from the sensor. ¨  ¨Pressing to maximum safe levels with minor discomfort to the wearer did not damage the sensors or saturate the data acquisition system, and thus, the sensors recorded the full range of treatment forces. ¨  ¨This simple and cost-effective method can be employed in a real therapeutic environment and that it is sufficiently reliable and sensitive to measure specific performance metrics for therapist skills assessments. Informal feedback from the therapists indicated that after a short period of acclimation to the feeling of the acrylic nail sensors, they were able to perform the clinical maneuver in the lab-based setting in a way similar to that of a real patient treatment. ¨  ¨Whether used clinically or as part of a training program, this quantifiable feedback may reduce the risk of adverse events. ¨ | ¨Interposed devices obstruct the therapist from the subject, which may confound application of the standard therapy protocols. A floor-mounted load cell setup would require precise tracking of the therapist’s point of load application. Thus, it may not capture tangential load components, or the net load vector may be attenuated by inconsistent positioning of the subject. The use of mannequins or instrumented dummies has been unable to reproduce the real experience of living humans. ¨  ¨For the novel device in this study, any interthumb difference in force applied during testing would increase error if it was not accounted for during calibration. ¨ |
| Lardon et al. (45) | To determine whether an 8-week physical exercise program could impact the SM learning among 1st-year chiropractic students. | Randomized controlled trial | **Patient-table interface:** same as in Triano et al., (23). | The authors report that this tool is valid and reliable in measuring force loads during manipulations (see Rogers and Triano (25)). | NA | NA |
| Lee et al. (29) | To measure the intervertebral movements of the cervical spine using iMR when it was subjected to PA mobilization. | Pilot study | **Clinician-patient interface:** A water-filled pressure device was employed to measure the PA force applied. A pressure pad was placed underneath the therapist’s thumbs which applied the PA force. This was connected to a clear plastic hose of internal diameter 5mm with one end open to atmospheric pressure. The pressure applied was measured by the height change of the column of water in the clear hose. | The pressure sensor had good linearity (force = 2.89 change in water column height - 18.99) and excellent correlation with known calibration force (r = 0.996). | ¨The present measurement system was found to be adequate for the force level generally experienced during cervical mobilization. ¨ | ¨In our pilot study, it was noted that the system was not able to accurately measure forces of greater than 70N due to the opposition of the two external surfaces of the pressure pad. ¨ |
| Mikhail et al. (14) | To quantify the difference between the forces measured at the patient-table interface and the force applied at the clinician-patient interface during standardized thoracic SMT and MOB in asymptomatic adults and to explore the factors related to SMT/MOB force characteristics and participant characteristics that potentially influence the magnitude of this difference. | Cross-over laboratory study | **Patient-table interface:** Same as Rogers and Triano (25). | The FSTT® has been shown to be reliable in the measurement of SMT force-time characteristics. (see Rogers and Triano (25)). | NA | NA |
| Petersen et al. (46) | To report both the reliability and accuracy of an expert physical therapist acting as a reference standard for a manual therapy joint mobilization trial. | Secondary analysis of a published randomized, controlled, crossover study. | **Clinician-patient interface:** Same as Petersen et al. (11). | NA | “Research has shown that when students are provided with objective feedback, they more consistently and accurately apply the desired manual therapy forces. Joint force measurement devices that provide quantitative feedback allow an expert clinician to share objective force parameters with students in a classroom setting. Flexible force monitoring devices that allow for direct force measurement between the therapist’s hands and the actual joint being mobilized have been shown to produce reliable and accurate measurements. When students are able to directly compare their performance on a joint mobilization skill to an expert standard, they can apply more accurate forces during manual therapy training.” | NA |
| Petersen et al. (11) | To investigate the effects of real-time, objective feedback using a direct force measurement device on entry-level students learning multiple grades and forces of lumbar spine mobilization. | Randomized controlled, crossover design | **Clinician-patient interface:** Small (10.5 × 5 cm), thin, load pad force monitoring device (Novel Electronics Inc., St. Paul, MN). | The authors report that the device can accurately record force magnitude, frequency, amplitude, timing, and rate of force production (Refers to previous studies). | ¨The device is portable, light-weight, flexible, and highly compliant, which provides minimal disturbance to the proprioception of the practitioner. ¨  ¨The findings demonstrate that this type of augmented feedback using a direct measurement device was beneficial in helping students perform more closely to a fellowship-trained physical therapist on some lumbar spine mobilization parameters. ¨  ¨Real-time, augmented feedback was beneficial to the learning and retention of the complex motor skill of lumbar spine mobilization.”  “However, when provided with objective feedback, student performance can become more accurate and consistent. ¨  ¨Devices that provide quantitative feedback allow an expert clinician to share objective force parameters with a multitude of students in a classroom setting. Our study supports the use of implementing similar devices into the teaching of joint mobilization, which can provide the learner with quantitative feedback on how to simulate expert practice. ¨  ¨The majority of subjects felt confident using technology which provided augmented feedback to enhance their learning. Additionally, 22 out of 24 subjects felt that the real-time feedback would help them learn and retain manual skills. ¨  ¨Real-time objective feedback using a direct force measurement device is beneficial for physical therapy students learning lumbar Spine joint mobilization. ¨ | NA |
| Rogers and Triano (25) | To evaluate the validity and fidelity of the Leander 900 Z Series treatment table with an imbedded AMTI force plate as a sensing system and to test its ability to quantify small, statistically significant changes in biomechanical parameters of spinal manipulative therapy. | Validation study | **Patient-table interface:** same as in Triano et al. (23). | **Accuracy:** the mean absolute differences from the input loads were 3.8N (2.1%) for static load release tests and 4.0N (2.2%) of dynamic tests. | ¨Such a system offers significant advantages in understanding manipulation biomechanics and determining the development of skill among developing manipulators. The ability to quantitatively estimate the directions of all loads acting through the body enhances the ability to create more realistic models of biomechanical effects. Knowledge of these directional components empowers researchers and instructors to identify and minimize undesirable vector components that might be harmful in some subpopulations of patients (eg, the elderly and those with comorbid pathology). It also allows investigations into means to optimize components considered useful in management of specific conditions.¨ | NA |
| Sheaves et al. (38) | To investigate the effects of frequency and self-control of feedback on physiotherapy students learning lumbar spinal mobilization. | Randomized controlled experimental study | **Patient-table interface:** same as in Snodgrass et al. (32). | The authors report that the equipment is highly accurate at measuring the vertical manual forces that were used in this study (mean SD absolute error, 1.1 1.5 N) (see Snodgrass et al. (33)). | NA | ¨The equipment is unique to this study and not generally available, which limits the generalizability of the results.¨ |
| Smit et al. (26) | To determine whether it is necessary to quantify the magnitude of force applied when teaching a grade I central posteroanterior (PA) mobilisation technique (according to Maitland) on the cervical spine. | Randomized controlled trial | **Clinician-patient interface:** Two independent variable resistance force transducers (FlexiForce^TM^, Tekscan, South Boston, MA). These ultra-thin (0.13mm) transducers are sufficiently flexible and pliable to allow for minimally intrusive force measurement. The chosen sensor (Model A101) has a measuring range of 0-500 gram. The sensor’s active sensing area has a round footprint with a diameter of 10mm which is large enough to measure the force exerted by the thumb and yet small enough to be placed side by side on the vertebra. | The sensors are very sensitive and accurately measure very low forces in grams. | ¨This tool can provide objective feedback to students for teaching purposes.¨  ¨The tool used in this study has the additional facility to give instant visual feedback on magnitude of force, amplitude and frequency of oscillation while the technique is being performed.¨  ¨It is an inexpensive, compact unit, which is easily set up, transported and stored. The computer program is uncomplicated and data is available instantly. The sensors proved to be highly durable since the same sensors could be used throughout this study. The small size and the versatility of the sensors make placement thereof easy. The sensors are very sensitive and accurately measure very low forces in grams.¨ | ¨The students’ feedback about the influence of the sensors on the technique indicated that 11 subjects (27.5%) reported a slight disturbance in tactile feedback, however, without a probable influence on the grading and the technique itself. Twenty-nine students (72.5%) found that the sensors had no influence.¨  ¨Five third year students and one fourth year student performed the technique with forces exceeding 500g, resulting in loss of data. The range of forces that can be measured by the tool can however be adjusted by changing the sensors. It is therefore recommended that the sensors could be changed to ensure that larger forces could also be measured in future studies.¨ |
| Snodgrass et al. (34) | To objectively quantify the cervical mobilisation force parameters applied by students following standard training, and to determine if manual force applications are consistent between students, and if they are affected by the characteristics of mobilised subjects such as gender or spinal stiffness. | Cross-sectional descriptive study | **Patient-table interface:** Same as in Snodgrass et al. (9). | Measurement accuracy and reliability have previously been reported as satisfactory, with a mean absolute error of 1.1N (standard deviation 1.5) in the vertical force direction and 3N in any force direction. (see Snodgrass et al., (32)). | ¨The instrumented table offers a means of providing feedback to students about their mobilisation forces, and quantifying how they apply forces after traditional instruction is the first step in designing learning strategies using this feedback.¨  ¨This method of measurement allows techniques to be performed as they would be clinically, without additional instrumentation between the hands of the student and the person being mobilised, therefore providing a more realistic environment for students.¨ | ¨The forces recorded by the instrumented table represent the total forces applied during the application of the cervical mobilisation technique, rather than the specific force being imparted to an individual spinal joint.¨ |
| Snodgrass et al. (35) | To compare cervical mobilization forces applied by physiotherapists and students, and the factors associated with forces for each group. | Cross-sectional descriptive study | **Patient-table interface:** Same as in Snodgrass et al. (9). | NA | ¨Real-time objective feedback could enable students to apply cervical mobilization forces that more closely match those of a practicing therapist.¨  ¨This method of measurement allows therapists and students to apply techniques as they would clinically, without additional instrumentation between their hands and the mobilized subject.¨ | ¨Using an instrumented table to measure forces means that global forces are recorded rather than the force generated at the interface between a therapist’s hands and a subject’s neck.¨ |
| Snodgrass et al. (36) | The primary aim of this study is to determine if students provided with real-time objective feedback can apply standardised cervical mobilisation forces. Secondary aims were to determine the effects of feedback on consistency between students, skill retention and student perceptions of learning. | Randomized controlled trial | **Patient-table interface:** Same as in Snodgrass et al (9). | The table has adequate accuracy (mean absolute error 1.1N (SD 1.5) in the vertical force direction, ≤3N in any force direction (see Snodgrass et al., (32)). | ¨This method enables students to practice as they would apply mobilisations clinically, without instrumentation between their hands and a subject.¨ | ¨Students must mobilise the same vertebral level with the spine identically positioned each session to standardise their practice and for retention tasks to be meaningful.¨ |
| Snodgrass et al. (33) | To establish the baseline mechanical properties of cervical spine mobilization and to determine if the applied forces are affected by the characteristics of therapists and mobilized subjects. | Randomized controlled trial | **Patient-table interface:** Same as in Snodgrass et al. (9). | Measurement accuracy and reliability have been previously reported as satisfactory, with the mean absolute error 1.1N {SD 1.5) in the vertical force direction and 3N or less in any force direction. (see Snodgrass et al., (32)). | ¨Using this method, therapists applied techniques as they would normally do clinically, without instrumentation between their hands and the mobilized subject that might have altered their usual technique.¨ | ¨The local forces transmitted to a vertebra could not be separated from the overall forces because of the use of an instrumented table for measurements.¨ |
| Snodgrass et al. (32) | To report the development of an instrumented treatment table and its calibration for the purpose of measurement of mobilisation forces applied to the cervical spine. | Validation and technical study | **Patient-table interface:** Same as in Snodgrass et al. (9). | **Accuracy**: Forces in the unloaded condition was very good for vertical forces (mean absolute error 1.1N, SD 1.5) and reasonably good for horizontal forces (2.8N, SD 2.4 for mediolateral, 3.4N, SD 1.5 for caudad-cephalad). In the loaded condition absolute error increased slightly for horizontal forces.  **Reliability**: ICC (2,1) values for forces recorded in each direction, and for both unloaded and loaded conditions in the horizontal directions, ranged from 0.99 to 1.00 (width of 95% CI ≤ 0.8 for any direction or condition). | ¨By using an instrumented treatment table to measure cervical mobilisation forces, the manual therapist is unencumbered by any instrumentation that might affect their application of force, and the clinical setting is effectively reproduced.¨ | ¨Evaluation of the current instrumented table indicated that forces recorded in the horizontal directions were not as accurate as those recorded in the vertical direction. This may limit conclusions about manual forces that are applied with large components of force at acute angles to the surface of the table.¨  ¨The forces recorded using an instrumented table can be affected by interaction with the patient.¨ |
| Snodgrass et al. (9) | To quantify the manual forces applied to the cervical spine during joint mobilization. | Cross-sectional descriptive study | **Patient-table interface:** Instrumented table consisting of a standard treatment surface fitted to a steel frame resting on 7 load cells (Xtran. Model S1W; Applied Measurement Australia. Sydney). Four load cells were positioned to measure vertical forces applied to the table (load cells 1-4; maximum load, 750N each), 2 load cells measured mediolateral force (load cells 5 and 6; maximum load. 350N each), and 1 load cell measured caudad-cephalad force (load cell 7; maximum load, 350N). | **Reliability:** It was found to be excellent, with tests in each direction yielding an ICC (2,1) of no less than 0.99, with confidence intervals no greater than 0.91 to 1.00 in any direction for either the preloaded or unloaded conditions.  **Accuracy:** It was found to be very good, with a mean absolute error of 1.1N (SD, 1.5). The mean absolute errors in the horizontal directions were slightly greater. With the table unloaded, the mean absolute error was 2.9N (SD, 2.4) in the mediolateral direction and 3.4N (1.5N) in the caudad-cephalad direction. When the table was preloaded, the mean absolute error increased for forces applied in the caudad-cephalad direction (10.7N; SD., 6.9) but made no real difference for mediolateral forces (3.9N; SD, 2.4). | ¨The instrumented table was used because it does not rely on any instrumentation between the therapist's hand and the subject that may alter the way a therapist applies manual forces.¨ | ¨The forces recorded by the table do not directly represent the manual force applied at the point of hand contact, but rather the forces transmitted by the subject's body to the table.¨  ¨When using an instrumented table, extraneous body movement of the subject on the table could potentially confound the force data.¨ |
| Starmer et al. (42) | To determine whether there was a change in peak force modulation and time-to-peak force in first-year chiropractic students following a 12-week detraining period from practice and feedback. | Pre-post descriptive study | **Patient-table interface:** same as in Triano 2002, now refered as the force-sensing table technology (FSTT). | Previous research (25) has demonstrated excellent reliability and validity of the FSTT in measuring force loads during manipulations. | NA | NA |
| Triano et al. (43) | To create an exploratory database of manipulation treatment force variability as a function of the intent of an experienced clinician sub-specializing in the care of children to match treatment to childhood category. | Cross-sectional descriptive study | **Patient-table interface:** same as in Triano et al. (23)**.**  **Clinician-patient interface:** A representative fingertip contact was applied to a triaxial load cell (ATI Industrial Automation) positioned on the back of the mannequin. | Excellent fidelity of the input forces was observed in the transmitted force (difference between force measured at both interfaces). The maximum attenuation at peak force ranged from 1.8% to 1.5%.  **Patient-table interface:** Authors refer to Rogers and Triano (25), and Triano et al. (13) for the methods used to ensure fidelity of measures. | NA | NA |
| Triano et al. (59) | To measure the progression of individual learner's ability, at different  stages during their education, to deliver force as they carry out high-velocity, low-amplitude (HVLA) procedures. | Cross-sectional descriptive study | **Patient-table interface**: same as in Triano et al. (23). | Fidelity in measure of input forces to the sensing table has been validated in  previous work (25). | NA | NA |
| Triano et al. (27) | To quantify elements of spinal manipulation therapy performance and to test the strategy of combined rehearsal and quantitative feedback as a means of enhancing student skill development for cervical and thoracic manipulative procedures. | Randomized, controlled study | **Patient-table interface:** same as in Triano et al. (23). | The authors mention that accuracy and feasibility to measure small changes in performance are reported elsewhere. | NA | NA |
| Triano et al. (23) | To quantify elements of performance of spinal manipulation and to test the strategy of combined rehearsal and quantitative feedback as a means of enhancing skill development. | Randomizedcontrolled study | **Patient-table interface:** An AMTI force plate was imbedded into a commercial treatment table (Leader 900 Z Series; Leader International, Port Orchard, Wash) to independently evaluate the biomechanical characteristics of loads acting through the patient’s body. | The authors mention that accuracy and feasibility to measure small changes in performance are reported elsewhere. | ¨The inverse dynamics method of recording loads passing through the subject’s body was selected because it offers significant advantages over direct measurement of applied loads. It is the only available system to obtain information about the total loads simultaneously acting through the patient including their direction and estimates of their amplitudes and speed.¨ | NA |
| Triano and Schultz (18) | To examine the differences in loads that pass through the lumbopelvic among three commonly used manipulation procedures. Effects of varying the posture of the patient during the treatment also were examined. In addition to quantifying the loads transmitted, the study tested the theoretical intent of the procedures in terms of direction and amplitude of load components. | In vivo biomechanical study | **Patient-table interface:** A specially constructed, split-surface treatment table was designed to support the patient while isolation the loads that reached the sensing surface to those that passed strictly through the plane transecting the torso into an upper and lower body segment at the lumbosacral junction. An AMTI (AMTI, Watertown, MA) force plate, capable of sensing forces and moments about all three axes, was mounted under the surface supporting the upper body above the transverse plane passing through the lumbosacral junction. | Validity and fidelity of table reaction loads were confirmed on comparison of the recorded amplitudes of forces and moments in the preliminary testing of suspended weights. Applied forces and moments were 88.7N and 39.0Nm, respectively, Reaction loads sensed by the force plate statically were 89.26N and 38.59Nm. The percent error in the dynamic recording amplitudes did not exceed 4.3%. | ¨The mechanical armature allowed the manipulator to perform realistically.¨ | NA |
| Tuttle and Jacuinde (37) | To describe the design, construction, and repeatability of measurements of [the] device and to assess the device against the stated design criteria. | Design and evaluation, technical  note | **Clinician-patient interface:** Flexiforce A201 sensors (Tekscan, Inc, South Boston, MA), which are rated as having a 1-lb (0.454 kg) range and a 9.53-mm-diameter active area were used. These pressure sensors are thin (0.2mm thick), low-cost whose resistance decreases with pressure applied on the sensor surface. Glued to 2 sides of the sensor are rounded silicone bumpers and/or metal washers to ensure that force is distributed over a constant area. | **Accuracy**: The mean ± SD difference between the applied load and the reading on the device was 0.20 ± 2.00N or 0.3% ± 14.9%. The limits of agreement that indicate how closely 95% of measurements would be expected to approximate the actual applied load were –3.8 to 4.2N or –29.5% to 30.1%.  **Repeatability:** The repeatability coefficient indicating the maximum difference between 95% of repeated measurements were ± 2.0N or 12.1%  **Calibration:** Conditioning is accomplished by applying an approximately 5-kg load to the sensor 5 times, for about 5 seconds each time. Calibration consists of a 1-point procedure using a mass of approximately 4 kg. The volume control of the computer is then adjusted until the digital readout corresponds with the applied load. | ¨It was easy to use and calibrate, with calibration taking less than a minute. ¨  ¨It cost less than $100 US dollars, having been produced for approximately $30 US dollars. ¨  ¨It allowed the techniques to be performed in as normal a way possible, as it could be placed between the patient and different parts of the therapist’s hand and was thin enough (3 mm thick if 2 washers were used and 8 mm thick if 2 bumpers were used) to be used with the therapist’s hands in normal positions for most techniques.  ¨It was able to provide real-time and archived feedback on force magnitude within 20% or 5 N for forces up to 45 N, with contemporaneous visual feedback and feedback from applied-force data stored for later evaluation. Its accuracy was within the design criteria, except that the percentage variability for forces below 5 N were greater than the specified 20%.¨  ¨It was either available off-the-shelf or could be constructed from readily available parts without specialist skills: the device was made from parts that were readily available from electronic suppliers and did not require specialist skills to construct.¨  ¨The described device provides a low-cost, practical method for measuring forces manually applied through 1 point of contact and is considered capable of providing useful feedback for students learning manual therapy skills. The device is able to assess forces applied at 1 point and is only accurate over a relatively narrow range of forces (5 to 45N). A maximum force of 45N was selected because the device was designed primarily for use with the cervical spine. ¨  ¨Positive feedback has been received on the device from both staff and students of undergraduate and postgraduate programs, as well as students after short continuing-education courses. Preliminary feedback from clinical educators, postgraduate students, and undergraduate students is that the device has been valuable in assisting students to more accurately modulate forces applied during assessment and treatment techniques. ¨ | ¨One strength of the device is that it directly measures the force between the user and the patient; however, as a result, it cannot measure net forces that include all points of contact. Nor can its measurements include contact between the user and patient that extends beyond the sensor, such as other parts of the finger, thumb, or hand. Compared to the device presented in this technical note, other devices used to provide feedback are more accurate and applicable over a larger range of forces; but these are at least an order of magnitude more expensive and interfere more with the performance of techniques. ¨  ¨The relatively slow response time of FSRs does not significantly affect their accuracy at loading rates commonly used in mobilization (less than 2 Hz), but could result in underestimation of force during more rapid techniques, such as high-velocity thrusts. ¨ |
| van Zoest et al. (30) | To investigate the merits of an evaluation method based on 3D force component data to measure passive manual contact forces exerted during a manual examination and treatment technique in a learning environment. | A multiple time-series experimental design | **Clinician-patient interface:** Same as in van Zoest et al. (24). | NA | ¨The hand palm-held sensor that was part of the measuring system allows for different techniques to be used on different parts of the body. ¨  ¨In conclusion, an evaluation tool using 3D contact force measurement allows the assessment not only of overall manual (force magnitude but also its directional aspect. Thus, such an evaluation is, from a biomechanical point of view, more complete and more valid. ¨  ¨Given the versatility of the measuring system, other manual examination and treatment techniques used on different parts of the human body of subjects and patients could be studied. ¨ | ¨The prototype measuring system used in this study has limitations in measuring actual clinical handling techniques, as has been previously noted. ¨ |
| van Zoest and Gosselin (28) | To evaluate the dynamics of chiropractic adjustments by 2 chiropractors using a hand/palm-held computerized 3-D direct force measuring system; and (2) to compare the measurement data and analysis results with the existing body of knowledge of chiropractic research. | Cross-sectional descriptive study | **Clinician-patient interface:** Same as in van Zoest et al. (24) | The 3-D force sensor was statically calibrated in 3 directions, and the 3-D force-measuring system was calibrated in z-direction. Their accuracy (validity and reliability) showed systematic respective random errors in the order of 1N or less. | ¨Both chiropractors that participated in this study reported that they felt quite at ease performing the adjustments with the instrument. Surprisingly, the chiropractors stated that they had no difficulty in producing tension at the levels of involvement and that their perceived thrusting patterns had not been significantly modified to achieve what they considered successful adjustments (perceived cavitation at all adjustments). ¨  ¨Subjects reported no discomfort during the procedures. The apparatus was warmed to body temperature between the chiropractor's hands to prevent unpleasant metal to skin contact. Subjects did not report any difference in receiving adjustments from hands only adjustments and adjustments with the apparatus.¨  ¨The prototype system as it exists right now could, with minor adaptations, be useful to practitioners and students to describe and develop their manual force perception and force-delivering skills by providing standardization and real time objective feedback regarding actions performed. The system could be used as an important tool in psychophysical perception research combined with feedback learning research. ¨ | ¨The rigid sensor housing does not allow for points of application as specific as a transverse or spinous vertebral process. Smaller and different shaped contact surfaces will have to be used. ¨  ¨For the description and analysis of a specific adjustment, adjustment-specific setups with more appropriate contact areas are needed. ¨ |
| van Zoest et al. (24) | To describe a hand/palm held computerized force measurement system for direct measuring of 3D manual contact force, including static calibration characteristics. | Validation study | **Clinician-patient interface:** The 3D force measuring chain consists of the following four components: (1) a 3D piezo-electric force sensor (Kistler varioCOMP multicomponent force sensor 9601A31, Kistler Instrumente AG Winterthur, Switzerland). (2) a charge amplifier (Kistler multichannel charge amplifier 5034A3, Kistler Instrumente AG). (3) an analog–digital converter (ADC) (Advantech ADC-card PCL-1800, Ad Vantech Co. Ltd. Taiwan). (4) a PC with data acquisition and real-time data presentation software (Labview 5.1, National Instruments, Austin TX,USA). The force sensor was positioned between the clinician’s hand and the participant’s skin. For in vivo testing the 3D force sensor is mounted, housed and preloaded with a bottom plate (26cm^2^),a segment of a sphere (maximal height: 27mm) and a centered preloading bolt with a ring. | It should be realised that accuracy is based on static sensitivity (slope of 0.99 in the 3 axes) and offset measures which deviate 61N. This probably explains the rather small deviations for reliability and validity measures. Drift measures tend to be better for the built-in force sensor in z-direction, especially in the condition of no load (drift was measured during a 300 s time-trial without and with a load (15 or 25 kg) on the force sensor and expressed through a least square regression line). Drift wwithout load varied between -0.01 and 2.42N, while drift with load varied -2.03 between -0.07N.  The smallest measurable output and output changes,0.26–0.48 N for the three force components are related to the set force and voltage ranges and the 12-bit resolution of the ADC-card (0.2441 N/bit). See Table 1 for specific values. | ¨The ADC card allows for kiloHertz sample frequencies, permitting more accurate force magnitude readings and cavitation recording. ¨  ¨The system can also be useful to practitioners and students, to describe and develop their manual force perception and force delivering skills by providing standardization and real-time objective feedback regarding actions performed. ¨ | ¨A rigid object of a defined size including contact surface (26 cm^2^) contact surface disturbs the practitioner–patient contact. ¨  ¨Practitioner’s haptic feedback during force delivery and perception of force vector characteristics might differ and be inferior to the situation with an unhindered or less disturbed patient–practitioner contact as is the case with existing measuring systems. ¨  ¨The contact area of the system is too large for its application to small joints or over complex vertebral regions via spinous or transverse processes. All this means that in its present form the prototype system has its limitations in measuring actual clinical handling techniques¨ |
| Waddington et al. (48) | To evaluate hand and back comfort in simulated therapists and patients to establish whether the tool is suitable for testing in the clinical environment as a hand/wrist injury risk control measure. | Cross-over descriptive study | **Clinician-patient interface:** Device reported in Waddington and Adams (31) but the hydraulic force transducer was replaced with an electronic force transducer. This modified version was referred to the MobDyn II. The MobDyn II was also modified by changing the working tip to one made of a softer molded rubber with a cupped section in the center of the tip for the spinous process (MobDyn IIb). | NA | ¨Current research suggests this tool is acceptably comfortable for the hands, and use of the readout on the tool can almost eliminate the variability of spinal mobilization forces. ¨  **¨**By changing to the softer, contoured rubber tip of the MobDyn IIb, the effective patient tool contact area was increased (706 mm^2^). Thus, forces were less concentrated and a more acceptable level of back comfort was achieved. ¨  ¨The modified spinal mobilization tool, MobDyn lIb, was found to be acceptably comfortable at the back and the hands. With a contoured soft rubber tip, the tool-comfort-cost was no longer significant between the sensitive thoracic spine and the lumbar spine. Furthermore, hand comfort was not significantly different between the tool and the pisiform grip. ¨  ¨The device could allow a risk control measure to reduce the exposure of hands to mobilization forces. ¨ | ¨The size of the prototype MobDyn was not optimal for use. ¨  **¨**Pisiform grip was significantly more comfortable than the MobDyn II. ¨  ¨Although the use of the softer tip on MobDyn lIb significantly decreased the "tool-comfort-cost" in simulated-patient back comfort, mobilizations with the pisiform grip remained more comfort able in both phases of the study. ¨ |
| Waddington and Adams (31) | To develop a manual mobilization device with maximum hand contact area, and to determine comfort in relation to the therapists’ application of a manual therapy technique using the pisiform grip. The second aim was to compare variability in attempts at standard force production made using a hand-held therapy device with and without instantaneous force readout being visible. | Technical study | **Clinician-patient interface:** The device reported in Waddington et al. 2006 was modified. A hand-grip strength dynamometer (Hand Dynamometer, Ausmedic Ltd., 4/37 Leighton Place, Hornsby NSW 2077, Australia) was modified. The grip handle of the dynamometer was reversed to create an internal oval shape in the device, to which a molded plastic handle and firm rubber tip were attached. | **Reliability**: In contrast, when three forces (50, 100 and 200 N) were applied 10 times to the digital scales, using the dial readout of the dynamometer to control force application, the ICC (2,1) was 0.999 (95% CI=0.996–0.9999). | ¨It is reported by the authors that integrating applied force readout into a mobilization device would enable the therapist to stop force application at a predetermined point. ¨  ¨It was found to be significantly more comfortable to use [the device] than the hands in the pisiform grip. ¨  ¨The surface area of the rubber tip of the device represents a trade-off between patient comfort and placement specificity. The area of the rubber tip was selected to be greater than the contact area of the average pisiform, but the selection is such that we are still able to place it over a single vertebra. ¨  ¨Manually applied force variability was significantly less and therapist comfort is greater when using a device with visual access to a dial giving immediate force readout. ¨ | ¨It is reported by the authors that embedding the therapy table in a force plate is likely to be too expensive a solution. ¨  ¨They also report that although instrumented plinths provide a functional research tool they do not provide a practical solution to determining mobilizing forces applied by the therapist in a clinical setting. ¨  **¨**In use the intervening dynamometer frame raises the height of the hands relative to the body, so are designed device, using and electronic force registration transducer rather than a hydraulic system, is needed to reduce device height. ¨ |
| Waddington et al. (47) | To develop a prototype tool capable of providing instantaneous feedback about manually applied forces, and to use it to determine (1) whether force constancy can be maintained during different application postures and (2) whether sensitivity to stiffness using the tool is different from sensitivity when assessing stiffness with the hands. | Two cross-over experimental studies | **Clinician-patient interface:** A prototype, "mobilizing dynamometer", adapted from a conventional lAMAR handgrip-strength dynamometer (Preston. Mich). A central feature is the integration of a calibrated force dial into the tool. As one of the components of the JAMAR, the force dial quantifies the magnitude of force produced during mobilization, allowing therapists to monitor force production. | Calibration of the dynamometer was checked against a set of Neopost TEP50 digital scales (Satas, France). A force was applied to the scale plate to values on the dynamometer dial between 50 and 400N, and upon reaching a specified force level, a reading was made simultaneously from the digital scales. Error never exceeded 1N for any force up to 400N. | ¨The molded handle allows the hypothenar eminence and metacarpal beads of the dominant hand to primarily sustain the force, whereas the wrist of that hand maintains a semi-extended position, and the free hand provides additional force and stability. Being large, the rubber indenter head helps increase surface area of contact between the tool and the patient's spine, a feature which has been found to be associated with greater patient-perceived comfort. ¨  ¨There was a large amount of variability both within and between subjects' ability to produce a consistent force. Using a device with a force readout has the potential to eliminate this variation. ¨  ¨It allows therapists to spread the forces sustained through the hands across a greater surface area of the palm and to spread the time of hand use over the day. It is anticipated that this reduced pressure will help protect therapists' hands and reduce the incidence of work-related wrist and hand injury.¨ | ¨Such devices raises the question of "loss of feel."¨  ¨It is also perceived that in using a tool to mobilize, patients lose the comforting sensation of the therapist's hands on their back. ¨ |

HVLA, high-velocity low-amplitude; PA, postero-anterior; LBP, low back pain; ROT, rotation; ICC, intra class correlation; SMT, spinal manipulative therapy; SM, spinal manipulation; DC(s), doctor(s) in chiropractic; iMR, interventional magnetic resonance; CI, confidence interval; FSTT, Force-sensing table technology.
