# Supplementary material for: Devices Used to Measure Force-Time Characteristics of Spinal Manipulations and Mobilizations: A Mixed-Methods Scoping Review on Metrologic Properties and Factors Influencing Use
Source: Front Pain Res (Lausanne). 2021 Oct 29;2:755877. doi: 10.3389/fpain.2021.755877 (PMC8915691; doi:10.3389/fpain.2021.755877)
Supplement: Supplementary file 2 [file Table_2.docx]

Supplementary Table 2. Metrologic properties reported for each device as well as the manual therapy modality evaluated and the targeted spine region. Numerical values and qualifiers are presented as stated in the included studies.

| Interface | # | Device and references | Modality (S or M) | Spine region | Metrologic properties | | | | | | | |
| --- | --- | --- | --- | --- | --- | --- | --- | --- | --- | --- | --- | --- |
|  |  |  |  |  | Measurement error (described as **V**alidity, **A**ccuracy, **F**idelity or **C**alibration) | **Rel**iability / **Rep**eatability | Coupling or cross-talk effect | Linearity / Correlation | Sensitivity | Variability | Drift | Calibration |
| Clinician-patient | i | Pressure pad with sensors  (7, 54-56) | S | C, T, SI | **A:** ± 7% of the actual value | NA | NA | NA | NA | NA | NA | NA |
|  | ii | Custom sensor worn on the nail  (44) | M | C | **A:** 0.17 ± 0.02N of force measured by load cell | NA | NA | NA | ≥0.02N | NA | NA | NA |
|  | iii | Water-filed pressure pad  (29) | M | C | NA | NA | NA | Good linearity (force=2.89 change in water column height - 18.99).  Excellent correlation with known force (r=0.996). | NA | NA | NA | NA |
|  | iv | Miniature three-dimensional force transducer  (10, 39-41, 43) | S | T, L | **A:** Good agreement (less than 3% differences) with a force measured by a force plate. | NA | NA | NA | NA | NA | NA | NA |
|  | v | I-Scan Sensor system (polyester sensing film)  (20) | S | T | **V:** Average error ≤ 3% of the force measured by a load cell. Absolute error for peak force ≤6%.  **A:** Accuracy reported in Rose et al. 1992. | NA | Small shear components. | NA | NA | NA | NA | Sensor equilibrated at 690 kPa (100 psi). Two-point calibration protocol with 345 and 690 kPa. |
|  | Vi | Two independent variable resistance  force transducers  (26, 57) | M | C | **A:** Accurately measure very low forces. | NA | NA | NA | Very sensitive. | NA | NA | NA |
|  | vii | Load pad force monitoring device  (11, 46) | M | L | **A:** Can accurately record force magnitude, frequency, amplitude, timing, and rate of force production (Refers to previous studies) | NA | NA | NA | NA | NA | NA | NA |
|  | viii | Pressure sensors  (37) | M | C | **A:** Mean (SD) of difference with known weights: 0.3 ± 14.9%. | **Rep:** Repeatability coefficient = 12.1% or 2.0N | NA | NA | NA | NA | NA | Sensors conditioned by applying a 5-kg load 5 times, for 5 s. Sensors calibrated using a mass of 4 kg. |
|  | ix | 3D contact force component measurement system  (24, 28, 30) | S, M | C, T, SI, table | **A:** Standard deviation of random error of <1N in the 3 axes with known weights. | **Rel:** Random error of <1N | NA | NA | Static sensitivity: slope of 0.99 in the 3 axes.  Smallest measurable output and output changes: 0.26–0.48N | NA | Without load: -0.01 to 2.42N.  With load: -2.03 to -0.07N | NA |
|  | x | Modified manual therapy dynamometer  (47, 48, 31) | M | T, L, plinth | **C:** Error never exceeded 1N for any force up to 400N | **Rel:** ICC(2,1) = 0.999 (95% CI=0.996–0.9999) for 10 repetitions of the same force. | NA | NA | NA | NA | NA | NA |
| Table-patient | xi | Adapted treatment couch with load cells  (8, 22) | M | L | **A:** Error of <2% with known weights | **Rel:** ICC [2,1], 99% CI = 0.99-1.0 | <2% | r^2^ = 0.9999 | NA | NA | NA | NA |
|  | xii | Custom-made instrumented treatment couch with load cells  (21) | M | L | **A:** Acceptable accuracy with error of ≤2.7% | NA | NA | NA | NA | NA | NA | NA |
|  | xiii | Standard mobilization couch adapted with load cells  (15-17, 19) | M | L | **A:** Each cell is accurate up to 150% of the 50-kg capacity. | **Rel:** Reliable over the range of forces applied.  Coefficient of variation <1%. | <2%. | High (r=0.99) for the 3 axes: r^2^=0,997 for x, and 0,999 for y and z. | < 1N along the Z-axis, and < 2N along X- and Y-axes.  20g in the vertical plane, 41g in the longitudinal plane and 74g in horizontal plane. | ≤2%. | <0.07% over a 10 min period | Forces were applied directly for z-axis calibration and via first  order pulley and weight systems for x- and y axes calibration. |
|  | xiv | Instrumented commercial table with force plate / Force-sensing table technology (FSTT^TM^)  (14, 18, 23, 25, 27, 41-43, 45, 59, 60) | S, M | C, T, L, SI, manikin | **A:** Mean difference ≤2.2% with known weights.  **F:** Excellent fidelity with maximum attenuation force ≤1.8%. | **Rel:** Excellent (refers to Rogers 2001) | NA | NA | Feasibility to measure small changes are reported elsewhere. | NA | NA | NA |
|  | xv | Instrumented table with load cells  (9, 32-36, 38) | M | C, L | **A:** Very good accuracy with mean absolute error 1.1N (SD 1.5) in the vertical direction and ≤3N in any direction. Measured with known weights. | **Rel:** Excellent with ICC (2,1) of ≥0.99 | NA | NA | NA | NA | NA | NA |
|  | xvi | Instrumented table with load cells  (20) | S | T | NA | NA | NA | NA | NA | NA | NA | NA |

Abbreviations: S, spinal manipulation; M, spinal mobilization or low force therapy; C, cervical spine; T, thoracic spine; L, lumbar spine; SI, sacroiliac articulation or sacrum; MDV, minimal detectable variation; SD, standard deviation; ICC, intraclass correlation; CI, confidence interval; N, Newtons; kPa, kilopascals; kg, kilograms; g, grams; s, second.
